# Asynchronous locking in metamaterials of fluids of light and sound

**DOI:** 10.1038/s41467-023-38788-9

**Published:** 2023-06-19

**Authors:** D. L. Chafatinos, A. S. Kuznetsov, A. A. Reynoso, G. Usaj, P. Sesin, I. Papuccio, A. E. Bruchhausen, K. Biermann, P. V. Santos, A. Fainstein

**Affiliations:** 1 Centro Atómico Bariloche and Instituto Balseiro, Comisión Nacional de Energía Atómica (CNEA) – Universidad Nacional de Cuyo (UNCUYO), 8400 Bariloche, Argentina; 2 Instituto de Nanociencia y Nanotecnología (INN-Bariloche), Consejo Nacional de Investigaciones Científicas y Técnicas (CONICET), Bariloche, Argentina; 3 Paul-Drude-Institut für Festkörperelektronik, Leibniz-Institut im Forschungsverbund Berlin e.V., Hausvogteiplatz 5-7, 10117 Berlin, Germany; 4 Departamento de Física Aplicada II, Universidad de Sevilla, E-41012 Sevilla, Spain; 5 TQC, Universiteit Antwerpen, Universiteitsplein 1, B-2610 Antwerpen, Belgium

**Keywords:** Semiconductors, Optofluidics, Bose-Einstein condensates

## Abstract

Lattices of exciton-polariton condensates represent an attractive platform for the study and implementation of non-Hermitian bosonic quantum systems with strong non-linear interactions. The possibility to actuate on them with a time dependent drive could provide for example the means to induce resonant inter-level transitions, or to perform Floquet engineering or Landau-Zener-Stückelberg state preparation. Here, we introduce polaromechanical metamaterials, two-dimensional arrays of *μ*m-sized traps confining zero-dimensional light-matter polariton fluids and GHz phonons. A strong exciton-mediated polariton-phonon interaction induces a time-dependent inter-site polariton coupling *J*(*t*) with remarkable consequences for the dynamics. When locally perturbed by continuous wave optical excitation, a mechanical self-oscillation sets-in and polaritons respond by locking the energy detuning between neighbor sites at integer multiples of the phonon energy, evidencing asynchronous locking involving the polariton and phonon fields. These results open the path for the coherent control of dissipative quantum light fluids with hypersound in a scalable platform.

## Abstract (web summary)

Exciton-polariton condensates are hybrid systems with nonlinear interactions. Here the authors demonstrate metamaterials with inter-site polariton coupling and asynchronous locking of light fluids from neighbor sites at the energy detuning.

## Introduction

Microcavity exciton-polaritons (simply polaritons, the quantum states formed by strongly coupled excitons and photons) constitute a hybrid system^1^ that displays a plethora of striking properties. These include Bose-Einstein condensation (BEC)^2^, superfluidity^3^, and Josephson-like oscillations^4,5^, with peculiarities stemming from the involved exciton-mediated repulsive Coulomb interactions and the driven-dissipative nature of the fluid^6^. The engineering of coupled pairs of polariton traps^4,5^ and arrays^7–9^ with controllable interactions^10^ has attained a degree of maturity that enables the implementation of quantum simulators^11–14^ and topological photonics^15^. Another emerging area is that of optomechanical crystals (OMXs)^16–19^, hybrid structures that bridge the optical domain with acoustics (in the MHz to GHz frequency range). OMXs exploit the Bragg co-localization of mechanical and optical modes to greatly enhance their interaction. Interestingly, cavity optomechanics has also been exploited to induce gauge fields as a resource for effectively breaking the time-reversal symmetry in topological photonics^20–22^ and phononics^23,24^. Polariton condensates are interesting for cavity optomechanical phenomena due to their long coherence times and the resonantly-enhanced exciton-mediated optomechanical coupling^25–30^. Both parameters, coherence time and coupling strength, are critical to boost the optomechanical cooperativity^18^. Photons and phonons follow the same wave equation in isotropic materials, and consequently the distributed Bragg reflector (DBR) planar microcavities hosting cavity polaritons in the near infrared also confine hypersound in the 20 GHz range^25^. The efficient interaction between these excitations has been shown to lead to a mechanical self-oscillation on continuous optical excitation^31^. The question then naturally arises: Can the powerful developments of cavity optomechanics be used in polariton systems relevant for optoelectronics and quantum technologies? Moreover: can the behavior of driven-dissipative light fluids be intertwined with coherent vibrations in a lattice to yield a collective behavior qualitatively different from that of its individual components?

**Fig. 1:**
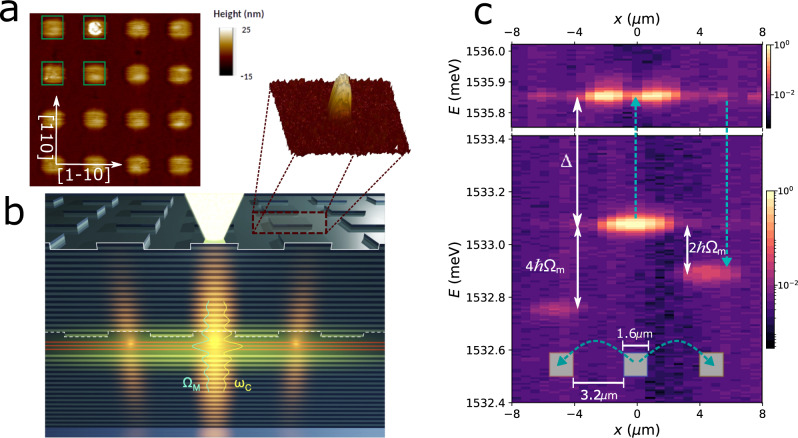
Polaromechanical metamaterials. **a** Is a 2D (top-view) atomic-force microscope (AFM) image of an array of 1 μm square intra-cavity traps separated by 1 μm-wide barriers. A scheme of the microcavity patterned with square-shape micrometer-size traps is presented in **b**. The three red lines in the spacer represent the quantum wells hosting excitons. The spacer thickness is larger at the position of the traps. Note that for clarity the scheme is not at scale: the real modulation of the spacer thickness is much smaller than a DBR period, for simplicity the DBR layers are represented flat, but in the actual structure they reproduce the shape of the microstructured spacer (the inset shows a top-surface AFM image of an individual 2 μm × 2 μm square trap). The laser is focused on one of the traps, and the neighbor sites are excited via the Gaussian tails of the laser spot, leading to coupled polaritons and vibrations in the individual unit cells of the array. **c** Shows the spatially resolved emission spectra obtained for such focused non-resonant excitation with powers above the polariton condensation threshold, for a square array of 1.6 μm square traps separated by 3.2 μm barriers. The central trap (*x* = 0) and the two closest neighbors (*x* ± 4.8 μm) can be identified. Both s-like symmetry ground (~1533 meV) and p-like excited (~1535.9 meV) states of polaritons in the traps are observed. Note that the neighbor trap ground state energies asynchronously lock at relative detunings corresponding to *δE* ~ − 2*ℏ*Ω_*m*_ and *δE* = − 4*ℏ*Ω_*m*_, where Ω_*m*_/2*π* ~ 20 GHz is the confined phonon frequency (*ℏ*Ω_*m*_ ~ 80 μeV). The dashed blue-arrows represent the optomechanically-induced inter-trap coupling mediated by virtual transitions to the excited state.

In this work, inspired by the idea of OMXs^16–19^, we go beyond the concept of Bragg structures and propose metamaterials based on resonant unit cells^32^. These consist of micrometer-size zero-dimensional intra-cavity polariton traps^8,33^, that confine and co-localize polaritons and acoustic vibrations (individual "polaromechanical” resonators), arranged into periodic arrays (see the AFM image of an actual array in Fig. 1a and the scheme in Fig. 1b). In these metamaterials, the on-site optomechanical interaction leads to a phonon-mediated time-dependent inter-site coupling *J*(*t*) of the trapped polariton condensates^34^. Striking signatures of the coherent polariton-phonon coupling emerge when the structures are optically driven with a continuous non-resonant and spatially localized optical excitation close to and above the threshold for condensation. Namely, the ground-states of polariton condensates at neighbor traps asynchronously lock at energies differing by integer numbers of the confined phonon energy, as illustrated in Fig. 1c. In order to explain this notable result we begin by describing the polariton and phonon bands of polaromechanical arrays. The characteristics of this locking phenomenon are then presented for different polaromechanical structures, and theoretically modeled in connection to synchronization and optomechanical phenomena. Conclusions are finally drawn on the potentiality of these results in the context of coupled non-equilibrium interacting exciton-polariton condensates.

## Results

### Polaromechanical metamaterials: co-localized polariton and phonon bands in lattices of 0D resonators

The proposed system is based on μm-sized traps previously studied in the context of polariton phenomena^8,31,33^, and created by micro-structuring the spacer of an (Al,Ga)As microcavity in-between growth steps by molecular beam epitaxy (MBE). Etching of the microcavity spacer prior to the growth of the top DBR into regions of smaller and larger thickness gives rise to optical and phonon cavity modes of higher or lower energy, respectively (see details in the Supplementary Notes 1 and 3). These provide the means to define traps and barriers in spatially tailored effective potentials. The magnitude of the effective potential modulation acting on the polaritons (typically some meV) is determined by the spacer etching thickness (typically around 10–15 nm) and by the cavity-exciton detuning. The etching is performed far from the quantum wells (QWs) so that the quality of the resulting excitonic system remains conserved. Arrays of polariton traps can also be fabricated with alternative technologies, as investigated by other groups (see e.g., ref. ^6^ and references therein). We note, however, that in order to display strong optomechanical phenomena as reported here, the embedded QWs need to be slightly displaced from the position of the maximum cavity optical field, which is the situation normally found in polariton microcavities^29^. At these latter positions, the strain associated with the confined phonon field is zero, and thus the exciton-mediated polariton-phonon coupling vanishes (see details in the Supplementary Note 1B).

**Fig. 2:**
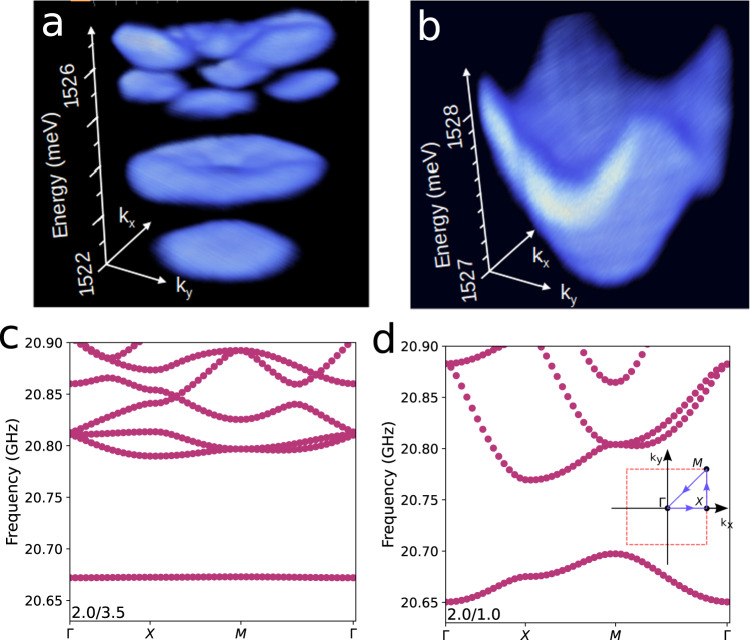
Polariton and phonon bands in lattices of 0D resonators. **a**, **b** Present the spectrally and wavevector-resolved polariton energies obtained by photoluminescence at low excitation powers in lattices of 4 μm × 4 μm and 1 μm × 1 μm square traps, respectively, both with 1 μm-wide barriers. The larger 4 μm × 4 μm traps display several confined modes, while only the ground state is confined for the smaller 1 μm × 1 μm structures. **c**, **d** Display the calculated in-plane dispersion of the acoustic modes of lattices of 2 μm × 2 μm square traps separated by 3.5 μm and 1 μm-wide barriers, respectively.

Panels a and b in Fig. 2 present the measured 2D polariton in-plane energy dispersion (i.e., along the *k*_*x*_ − *k*_*y*_ plane) for a 7 × 7 array of 4 μm × 4 μm square traps and a 10 × 10 array of 1 μm × 1 μm square traps, respectively, in both cases separated by 1 μm barriers. These dispersions were measured by angular-resolved photoluminescence (PL) with low optical powers (i.e., well below the BEC threshold). The full array was illuminated with a large spot of ~ 50 μm diameter. The larger 4 μm × 4 μm traps confine several states with energies below the finite barriers^33^. When organized in arrays this results in the formation of several bands, as displayed in Fig. 2a. The lower energy band derives from trap states of s-like symmetry, and is comparatively flat due to the larger degree of confinement (i.e., lower hybridization with neighbor traps) of these states. In contrast, the smaller 1 μm × 1 μm traps only confine the ground state (Fig. 2b) . Note that due to the smaller size, in this latter case the ground state is closer to the barrier edge. Consequently, the hybridization is larger resulting in a broader band (when compared to the 4 μm × 4 μm case). The reciprocal space dispersion in this case resembles the one for dispersive electron bands in a tight-binding model, where the role of the atomic electron level is played by the discrete polariton ground state of the 0D traps.

As mentioned above, in planar microcavities vibrations are also confined^25^, with a fundamental frequency around ${\Omega }_{m}^{0}/2\pi \sim 20$ GHz corresponding to a breathing of the cavity spacer along the growth direction, and overtones at ${\Omega }_{m}^{n}=(1+2n){\Omega }_{m}^{0}$. The wavelength of the fundamental confined mode is the same as the one for the confined photons (which is determined by the cavity spacer thickness). The frequency difference (from tens of GHz for the phonons to hundreds of THz for the photons) just bears the relation between the respective wave velocities. The local etching of the spacer thickness blue-shifts the phonon mode energy in the same proportion as for the confined photons (see Supplementary Note 4) and, consequently, an effective lateral potential develops for the confined acoustic phonons as it does for the polaritons. The calculated phonon dispersion around the Γ point of the Brillouin zone (*k*_*x*_ = *k*_*y*_ = 0) for arrays of 2 μm × 2 μm traps with ~3.5 μm and ~1 μm-wide barriers are presented in Fig. 2c, d, respectively (the model used is described in the “Methods” section). Phonon bands arise for the 2D lattice of traps and, mimicking what was observed for the polaritons, the lower energy band is flatter for the case of more isolated ground states (Fig. 2c). Note that, as for the polariton dispersions presented in Fig. 2a, b, the width of the bands can be tuned by the size of the traps, while for the calculated phonon bands in Fig. 2c, d the same is accomplished by changing their separation.

### Experimental study of phonon confinement in traps

**Fig. 3:**
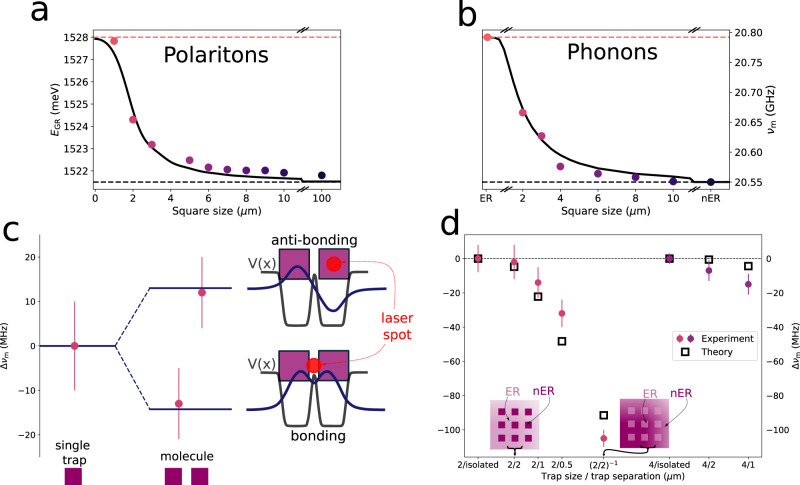
Experimental study of confined phonons in traps and lattices of traps. **a** Presents the measured size dependence of the energy of the polariton ground confined mode in isolated square traps. **b** Similar to **a** but for the phonon ground state. In both panels the limits of the induced potential, corresponding to planar etched (ER, barrier) and non-etched (nER, well) regions, are indicated by the dashed horizontal lines. The continuous black curve is the effective potential model. The case for a molecular-like phonon double-trap made of two 2 μm × 2 μm traps separated by 0.5 μm, is illustrated in **c**. The phonon potential, and the calculated bonding and anti-bonding-like levels are shown, together with a scheme of the respective excitation condition at different sites. Symbols correspond to the experiments, while the solid lines are the theoretical predictions. **d** Summarizes the phonon experiments on arrays of traps of 4 and 2 μm lateral size, with different inter-trap separation, and their comparison with the effective potential model. Again, the energies are given with respect to that of the corresponding single trap (identified as “2/isolated” and “4/isolated” in this panel). The arrays are labeled as *a*/*b*, with *a* the trap size, and *b* the inter-trap separation. The case of an inverted array of 2 μm etched regions separated by 2 μm non-etched regions (labeled as (2/2)^−1^ is also included. Circular full (square empty) symbols are the experimental (theoretical) values. Error bars in **c** and **d** indicate the standard deviation of ten spectral measurements.

Figure 3 expands on an experimental study of the phonons confined in individual square traps, molecule-like double-traps, and in arrays of traps. Panels a and b in Fig. 3 present a comparative study of the polariton and phonon ground-state energy for isolated traps of varying square size. The polariton energies in panel a were obtained from PL data recorded at 5 K under low excitation power (i.e., well below BEC)^33^. The trap spectra correspond to discrete levels, the number of confined states depending on the size of the traps (only data for the ground state are presented in Fig. 3a). The limits of the induced potential, corresponding to measurements performed on planar etched (barrier) and non-etched (well) regions are indicated by the dashed horizontal lines. The expected size-dependence for a trapping potential with finite barrier height is observed. The experimental data are compared in Fig. 3a with a theoretical calculation based on an effective potential model with a realistic description of the trap potential^33^ (black solid curve, see Supplementary Note 4D for details).

To experimentally study the high frequency vibrations in the same traps, we used a picosecond coherent phonon pump and probe technique^35–37^. A ps-laser pulse is used to resonantly excite the optical cavity mode, generating coherent phonons by a displacive mechanism. These mechanical oscillations in turn modulate the cavity energy, thus allowing their detection using a delayed probe pulse. Traps were individually addressed using a microscope set-up with a ~3 μm-wide Gaussian spot (more details are provided in Supplementary Note 2). The dashed horizontal lines in Fig. 3b represent the measurements in extended planar non-etched and etched regions, respectively. These define, as for the polariton case discussed above, the limits of the induced lateral phonon effective potential (traps and barriers, respectively). Within these limits the phonon trap energies increase with decreasing size, as expected for confined states in a trap with finite barriers (examples of experimental spectra are provided in the Supplementary Note 3). The observed shift is very well described by a phonon effective potential model (black solid curve) based precisely on the same parameters for the traps as used to describe the polariton energies (see the “Methods” section). The similarity with the behavior of polaritons in Fig. 3a emphasizes the concept of polaromechanical traps in which both polaritons and phonons are confined in 0D resonators. We have detected confined polaritons and phonons in traps with dimensions down to 1 μm × 1 μm, exhibiting record coherence times for polariton condensates (ns-long) and for confined phonons (100’s ns) with no observable reduction with decreasing trap size. We note, in contrast, that a study of cavity confined phonon dynamics in etched micropillars^38^ showed a significant decrease of the mechanical mode lifetime for pillar diameters below 7 μm.

We now turn to architectures of coupled 0D resonators. Experiments demonstrating the formation of polariton bands were described in Fig. 2a, b, we concentrate here on the phonon properties. The case of a molecule-like structure made of two 2 μm × 2 μm traps separated by 0.5 μm is presented in Fig. 3c. The symbols correspond to the experimental values, and the horizontal lines are the calculated energies. To selectively excite each state we positioned the laser spot either symmetrically between the traps, or on top of one of them (as shown in the scheme of Fig. 3c). Two states of anti-bonding and bonding character arise, split relative to the individual trap by the interaction energy ± *J*. The modeled effective phonon potential corresponding to the double-trap structure is also shown, together with the calculated spatial shape of the resulting bonding and anti-bonding states. The measured splitting of the modes, and their ordering (bonding state at lower energy), are well described by the theory.

Figure 3d summarizes experiments on a series of arrays of square traps of 2 μm and 4 μm lateral size, and different inter-trap distance. These are labeled as *a*/*b*, where *a*(*b*) identifies the trap size(separation) in μm. An “inverted” array is also included, corresponding to etched squares of 2 μm × 2 μm size, separated by 2 μm-wide non-etched channels [labeled as (2/2)^−1^]. All frequencies are given with respect to that of the respective isolated single trap. The measured frequencies are also compared to those calculated for the Γ point “s”-like vibrations (i.e., the lower energy mode at *k*_*y*_ = 0 in Fig. 2c, d), shown with open squares in Fig. 3d. Due to the experimental geometry with light incident within a small cone around the normal direction, the probe pulse couples with *k* ~ 0 vibrations. Since, in addition, the spatial distribution of the Gaussian pulse is uniform at the scale of an individual trap, it is sensitive to the more symmetric “s”-like ground state. Note that the observed red-shift of the detected modes with decreasing inter-trap separation (e.g, from 2/2 to 2/0.5) is a measure of the array phonon half band-width (*J*). For inter-trap separations ≳2 μm the Γ point mode is almost coincident in frequency with that of the individual trap, signaling the flat-band limit. Note also the weaker red-shift of the Γ point mode of the 4 μm × 4 μm trap lattices, when compared to those constructed from 2 μm × 2 μm traps, reflecting the relatively flatter-band nature of the former. Indeed, as for the polariton bands in Fig. 2a, for larger traps the on-site energies are smaller and thus red-shifted farther down from the barrier edge. Consequently, the states become less delocalized when traps are coupled in an array.

### Driven polaromechanical crystals: asynchronous energy locking

Having established the properties of phonon and polariton excitations in the proposed polaromechanical metamaterials, we turn now to the main result of our work, i.e., the observation of a locking of the inter-trap polariton energy detuning at fixed differences that correspond to integer numbers of the phonon energy. This occurs when the in-plane polariton dynamics in these polaromechanical metamaterials is induced by optically driving the traps with micro-focused non-resonant continuous-wave optical excitation, with powers close to and above the threshold for BEC. A Gaussian shape exciton reservoir is formed, with a size somewhat larger but of the order of the spot size (typically ~3−5 μm in our experiments). As this problem is presently understood in the context of synchronization phenomena in polariton condensates^39–43^, the related Coulomb repulsion, together with residual disorder, and the inter-trap coupling *J*, are expected to determine the local energies (and thus the dynamics) of polaritons in the traps. When polariton traps are coupled (large *J*), the Josephson flow is responsible for the phase locking between the condensates (synchronization)^39–41^. If the potential difference between the traps (determined by local disorder^42,43^ and eventually by an inhomogeneous Coulomb interaction^44^, as is the case for our Gaussian shape excitation) exceeds a certain critical value (limit of small *J*), the Josephson flow cannot reach a steady state and the condensates cannot synchronize. Quite notably, however, as shown in Fig. 1c for an array of 1.6 μm × 1.6 μm traps separated by 3.2 μm barriers, the neighbor site polariton condensate ground state energies in our experiments neither follow a simple Gaussian distribution (as would be expected for uncoupled polariton condensates) nor share the same frequency (as would happen if they synchronize), but seem to lock at detunings that correspond to integer multiples of the mechanical phonon energy *ℏ*Ω_*m*_ - the asynchronous locking.

**Fig. 4:**
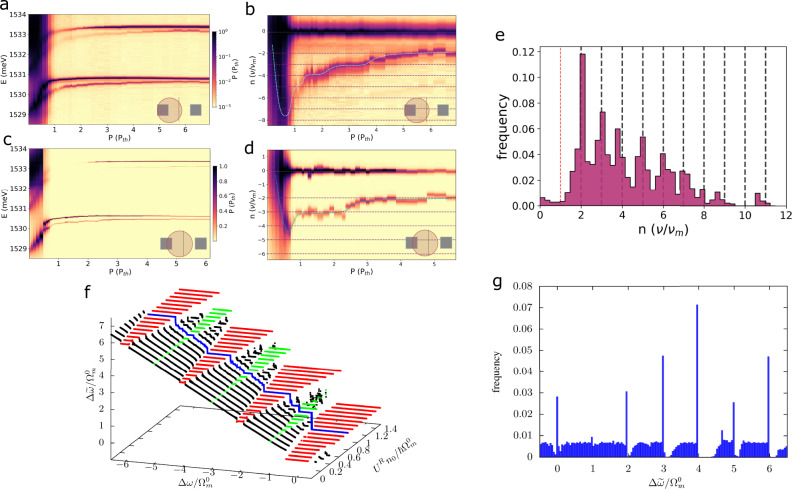
Polaromechanics: asynchronous locking of weakly coupled traps. **a**, **c** Display the excitation power dependence of the polariton emission for an array of square traps of 1.6 μm lateral size separated by 3.2 μm-etched regions, and for two different spot positions (indicated at the bottom-right of each figure). The detuning between the ground states of the two neighbor traps is presented in **b** and **d**. In these panels the more pumped trap defines the zero of energy, and the detuning is given in units of ${\nu }_{m}^{0} \sim 20$ GHz, the fundamental cavity phonon mode. A histogram on the frequency of occurrence of the different detunings, obtained from an extensive series of experiments in arrays of 1, 1.3, and 1.6 μm square traps separated by 2, 2.6, and 3.2 μm barriers, respectively, is presented in **e**. **f** Shows the dressed detuning $\Delta \widetilde{\omega }$, obtained numerically using the model described in the Supplementary Note 8B, with two phonons of frequencies ${\Omega }_{m}^{0}/2\pi=20$GHz and ${\Omega }_{m}^{1}/2\pi=60$GHz, as a function of the bare detuning Δ*ω* and the reservoir-polariton interaction *U*^*R*^*n*_0_. Regions where frequency locking occurs (at $n{\Omega }_{m}^{0}$) are shown in red (for even values of *n*) and in green (odd values of *n*). The blue line highlights the behavior of the detuning for a given value of the interaction ${U}^{R}{n}_{0}=0.8\hslash {\Omega }_{m}^{0}$ (to be compared with **b** and **d**). **g** Presents a histogram of dressed detuning obtained using a self-consistent model for the phonons (see text and the Supplementary Note 8C). Note the emergence of peaks at integer values of *n*, as in the experimental histogram in **e**.

Figure 4a–d present two examples from an extensive study performed in an array of 1.6 μm × 1.6 μm traps separated by 3.2 μm barriers. In these experiments careful micro-positioning and a tight focus of about 3 μm was used to locally excite different traps of the array, and at different positions between neighbor traps of the array. Two representative cases are shown: in Fig. 4a, b the excitation is strongly unbalanced with the spot positioned almost on top of one of the traps, while for Fig. 4c, d the excitation is also unbalanced but the spot is closer to the midpoint between the traps (the complete series of experiments for varying position between these two traps can be found in Supplementary Note 7D). In Fig. 4a, c the emission intensity is presented as a function of energy and excitation power *P* (the latter in terms of the condensation threshold power *P*_*th*_). At low powers, the ground states of the two traps are observed close to 1.529 eV, while the first excited states appear at ~1.531 eV (the corresponding spatial images are very similar to the one in Fig. 1c). Condensation is signaled by the narrowing of the modes (note that also the excited states become macroscopically occupied at higher powers). Figure 4b, d detail the power dependence of the ground states, referencing the energy to the one of the pumped trap, and by measuring the inter-trap detuning in units of the fundamental confined phonon frequency ${\nu }_{m}^{0} \sim 20$ GHz. As expected, for *P* < *P*_*th*_ the pumped trap blue-shifts due to interaction between polaritons and with the reservoir. The neighboring trap, not being directly pumped, shifts less, thus explaining the initial steep increase in detuning (the magnitude of the latter is directly determined by the unbalance of the laser spot position). Then for *P*/*P*_*th*_ > 1, owing to gain saturation of the pumped trap, the neighbor trap catches up, thus reducing the detuning. The asynchronous locking shows up in this figure as a staircase plot with flat sections at integer numbers of $n{\nu }_{m}^{0}$.

We have observed a similar locking behavior also in an array of smaller 1.3 μm × 1.3 μm traps separated by 2.6 μm barriers, and in another one of very small and closely packed 1.0 μm × 1.0 μm traps separated by 2.0 μm barriers (the corresponding data are presented in Supplementary Notes 7E, F, respectively). Flat (locked) regions appear more or less pronounced in different experiments. An alternative way to evidence the locking is through a histogram counting the frequency of occurrence of a certain detuning, where flat sections should appear as peaks. Such plot is presented in Fig. 4e, for the collection of some 10 different experimental runs performed at different laser positions and in the three studied trap arrays (details on how this histogram is obtained can be found in Supplementary Note 7G). Evenly spaced peaks appear at integer numbers of $n{\nu }_{m}^{0}$ (up to *n* = 7), providing compelling evidence for the emergence of the asynchronous locking.

**Fig. 5:**
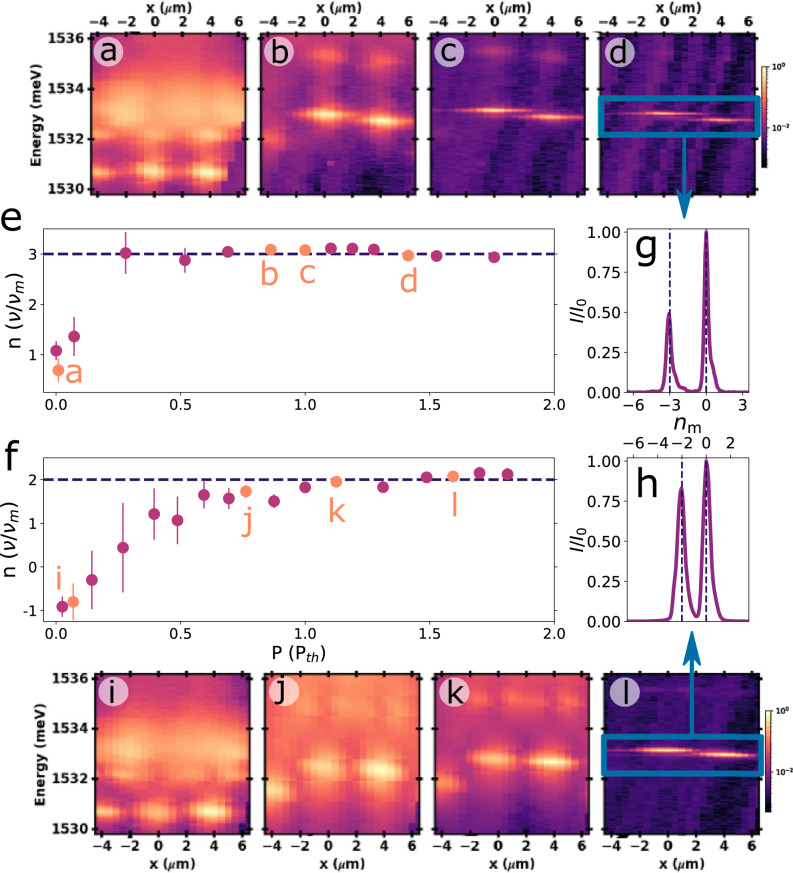
Polaromechanics: asynchronous locking in the finite *J* limit. Excitation power dependence of the polariton emission spectra for an array of square traps of 1.3 μm lateral size separated by 2.6 μm- etched regions. Examples of energy-resolved spatial images for some selected pump powers are shown in **a**–**d** for a row of traps along the crystal direction Y [-1 -1 0] (see the AFM image in Fig. 1a). The spectrum corresponding to high-powers is displayed in **g** (the vertical dashed line indicates an integer number of phonon quanta). A similar sequence of spatial images and high-power spectrum corresponding to a row of traps along the orthogonal direction X [−1 1 0], are shown in **i**–**l**. The spectrum corresponding to high-powers (**l**) is displayed in **h**. **e**, **f** summarize the frequency detuning between the pumped and a neighbor trap as a function of pump power (given in terms of *P*_*th*_, the condensation threshold power), for the two studied cases. The symbols corresponding to the shown spatial images are differentiated with orange color. The error bars indicate the standard deviation of ten spectral measurements.

A qualitatively different situation can be investigated if now the laser excitation is more uniform, and this is presented in Fig. 5 again for the square array of 1.3 μm × 1.3 μm square traps separated by 2.6  μm barriers. In this case the pupil of the microscope objective (of numerical aperture NA=0.3) was under-filled to obtain a larger spot size of ~ 5 μm, leading to a still unbalanced but more uniform excitation of pairs of traps. Figure 5a–d present energy-resolved spatial images for increasing non-resonant excitation power, obtained from one row of traps aligned along the crystal direction Y [-1 -1 0]. At very small powers, three traps characterized by relatively broad lines are observed approximately at the same ground state energy (the central trap is at *x* = 0 μm, the closest neighbors appear at ~± 4 μm). As the power increases three of the traps blue-shift (panels a–c), as expected from the Coulomb repulsion with the exciton reservoir, the left-most remaining farther away in energy. This asymmetry between left and right traps arises from a slight shift of the laser spot towards one of the neighbors. As the modes shift with increasing pump power, a clear line-narrowing and non-linear increase of intensity signals the transition to the condensation of the light fluid above *P*_th_. At the highest powers, again the blue-shift saturates. Note that the energy of the neighbor trap that also blue-shifts gets rapidly locked with increasing power at a detuning that corresponds to three times the phonon frequency (see the high-power spectra displayed in Fig. 5e). Figure 5f–i display the corresponding spatial images from the same array, but now for a row of traps aligned along the orthogonal crystal direction X [−1 1 0] (square traps grow with a slight rectangular asymmetry, so that the coupling *J* of the ground states along Y is somewhat smaller than along X^33^). As evidenced by the high-power spectrum in Fig. 5j, the neighbor trap detuning locks again at an integer number of the phonon energy quanta (two in this experiment). The full power dependence of the detuning between the most intense neighbor trap and the central one for the two reported cases is shown in Fig. 5e (along Y) and f (along X). Quite clearly again an asynchronous locking is observed. In contrast to the experiments in Fig. 4, however, with the more uniform excitation the initial overshoot of the inter-trap detuning is avoided thus facilitating the locking.

### The model

The studied arrays span the parameter space from a situation in which the direct Josephson coupling *J* between ground states is negligibly small (the 1.6 μm-trap arrays), to another in which the opposite is true (the 1.0 μm-trap array), implying the universality of the observed locking phenomenon. A realistic description of the 1.6 μm × 1.6 μm traps separated by 3.2 μm barriers (data displayed in Fig. 1c and 4) using the Gross-Pitaevskii model shows that the overlap integral between ground states of neighbor traps is negligible (~10^−4^), representing the flat-band limit described above (see the analysis in Supplementary Note 6A). On the other extreme, the same modeling for the 1.0 μm × 1.0 μm traps separated by 2.0 μm barriers, shows that the ground states are close to the top of the barriers with large inter-site overlap, thus representing the highly connected limit. The 1.3 μm × 1.3 μm traps separated by 2.6 μm barriers fall somewhere in between, with a finite *J* that is a fraction of the confined phonon frequency. Interestingly, even for the case in which the direct *J* ~ 0, it can be shown^34^ via second-order perturbation theory that a phonon-induced inter-site quadratic optomechanical coupling *g*_2_ arises, involving virtual transitions between the isolated polariton ground states and an extended p-like excited level at energy *ℏ*Δ above the ground state (see the scheme in Fig. 1c and the Supplementary Note 6). For small traps, such as those leading to the locking behavior shown in Figs. 1c and 4, the involved p-like state at *ℏ*Δ ~ 2.8 meV is indeed shared by both sites. For this case it follows that ${g}_{2}={g}_{0}^{2}/\Delta$ (here *g*_0_ is the on-site linear optomechanical coupling)^34,45^. The concept of a polaromechanical metamaterial is, thus, also relevant in this regime, because the polariton inter-trap coupling is fully determined by the on-site optomechanical interactions of the hybrid metamaterial resonant unit-cell.

Our observations are related to the physics of synchronization, which is intrinsic to non-linear dissipative dynamical systems such as the coupled polariton condensates^39–43^. We extend the above to the case of a mechanically-modulated time-dependent inter-trap coupling. To model the observed physics we follow Wouters^39^, and describe two coupled polariton modes and the corresponding reservoir as:

1 $$i\hslash \dot{{\psi }_{j}}=	\left({\varepsilon }_{j}+{U}_{j}|{\psi }_{j}{|}^{2}+{U}_{j}^{R}{n}_{j}\right){\psi }_{j}-J{\psi }_{3-j}+\frac{i\hslash }{2}(R{n}_{j}-\gamma ){\psi }_{j},\\ \dot{{n}_{j}}=	{P}_{j}-{\gamma }_{R}{n}_{j}-R|{\psi }_{j}{|}^{2}{n}_{j}.$$

Here *ε*_*j*_ is the bare energy of the *j*-mode (*j* = 1, 2), *U*_*j*_ and ${U}_{j}^{R}$ are the polariton-polariton and polariton-reservoir interaction couplings, *J* describes a hopping term between modes, *γ* the polariton decay rate and *R* the stimulated loading from the reservoir. The dynamic of the latter is controlled by the pump power *P*_*j*_, the excitonic decay rate *γ*_*R*_ and the stimulated decay to the condensate.

While in ref. ^39^ *J* was considered a constant, in our systems all cavity parameters are intertwined with well-defined phonon modes so we expect *J* to be modulated by the phonon field, that is, *J*(*x*_*n*_) with *x*_*n*_ the generalized coordinate of the corresponding phonon mode. This naturally leads to a time-dependent coupling when phonon oscillations are present. When the traps are relatively close, single phonon process might occur and hence a linear coupling is expected. On the contrary, when the traps are far apart, the second order process discussed above enters into play and a quadratic coupling is obtained. Before considering the time dependence, let us first revisit the static case. We then seek for a solution of the form^39^: ${\psi }_{j}=\sqrt{{\rho }_{j}}{e}^{-i\omega t}{e}^{\pm i\theta /2}$, that is, two states with a synchronized identical frequency *ω* (here the + sign corresponds to *j* = 1) and $\dot{{n}_{j}}=0$. After some algebra, and taking *R*∣*ψ*_*j*_∣^2^ > > *γ*_*R*_, one finds that synchronization occur whenever there is a solution for *θ* that satisfies $\Delta \bar{\varepsilon }(\theta )=J\cos \theta (\alpha {(\theta )}^{-1}-\alpha (\theta ))$ with: $\Delta \bar{\varepsilon }(\theta )={\bar{\varepsilon }}_{2}(\theta )-{\bar{\varepsilon }}_{1}(\theta ),{\bar{\varepsilon }}_{j}(\theta )={\varepsilon }_{j}+{U}_{j}^{R}{n}_{0}\,{\xi }_{j}^{0}/{\xi }_{j}(\theta )+{U}_{j}{\rho }_{0}\,{\xi }_{j}(\theta ),\alpha (\theta )=\sqrt{{\xi }_{2}(\theta )/{\xi }_{1}(\theta )}$, and we have introduced the dimensionless parameters so that *ξ*_*j*_ = *ρ*_*j*_/*ρ*_0_, *ρ*_0_ = *γ*_*R*_/*R*, and *n*_0_ = *γ*/*R*—the equations for *ξ*_*j*_(*θ*) are given in the Supplementary Note 8A. While a solution exists even in the absence of interactions (when *ε*_2_ − *ε*_1_ ≤ 2*J*^2^/*γ*), it turns out that the presence of *U*_*j*_ and ${U}_{j}^{R}$ strongly favors the appearance of a synchronized phase (see e.g., Fig. 1 of ref. ^39^).

We now consider a coupling that is quadratic in the phonon displacement. Hence $J(t)={J}_{m}({e}^{i2{\Omega }_{m}t}+{e}^{-i2{\Omega }_{m}t}+2)$ with *J*_*m*_ = *g*_2_*n*_*b*_. This follows from assuming the presence of a coherent population of *n*_*b*_ phonons, i.e., $b(t)+b{(t)}^{*}=2\sqrt{{n}_{b}}\cos ({\Omega }_{m}t)$, and an optomechanical interaction having a hopping term between the two polariton modes with a prefactor containing the second-order power of the phonon displacement, namely, $-\hslash {g}_{2}{(\hat{b}+{\hat{b}}^{{{{\dagger}}} })}^{2}$ (see Supplementary Note 8). In the rotating wave approximation (RWA), assuming *ε*_1_ ~ *ε*_2_ + 2*ℏ*Ω_*m*_, keeping only the resonant terms, we have

2 $$i\hslash \dot{{\psi }_{1}}=	{\bar{\varepsilon }}_{1}{\psi }_{1}-{J}_{m}{e}^{-i2{\Omega }_{m}t}{\psi }_{2}+\frac{i\hslash }{2}(R{n}_{1}-\gamma ){\psi }_{1},\\ i\hslash \dot{{\psi }_{2}}=	{\bar{\varepsilon }}_{2}{\psi }_{2}-{J}_{m}{e}^{i2{\Omega }_{m}t}{\psi }_{1}+\frac{i\hslash }{2}(R{n}_{2}-\gamma ){\psi }_{2},$$

with ${\bar{\varepsilon }}_{j}={\varepsilon }_{j}+{U}_{j}|{\psi }_{j}{|}^{2}+{U}_{j}^{R}{n}_{j}$. By comparison with Eq. (1) above it follows that now solutions can be found proposing ${\psi }_{1}=\sqrt{{\rho }_{1}}{e}^{-i\omega t}{e}^{i\theta /2}$ and ${\psi }_{2}=\sqrt{{\rho }_{2}}{e}^{-i(\omega -2{\Omega }_{m})t}{e}^{-i\theta /2}$, i.e. two condensates with frequencies locked at a fixed detuning given by 2Ω_*m*_^46^. The resulting synchronization conditions are the same as in the static case except that now *ε*_2_ → *ε*_2_ + 2*ℏ*Ω_*m*_. Consequently, all the conclusions derived for synchronized polariton condensates also apply here, except that synchronization is now represented by the mechanically induced asynchronous locking of the polariton condensate energies. This simple analysis in the RWA captures the essence of the full solution. This is shown in Fig. 4f where we plot, for the complete model beyond the RWA, the frequency detuning between the two polariton modes $\Delta \widetilde{\omega }$ [obtained by looking at the maximum peak of ${\psi }_{1}(\widetilde{\omega })$ and ${\psi }_{2}(\widetilde{\omega })$] as a function of the bare detuning, Δ*ω* = (*ε*_2_ − *ε*_1_)/*ℏ*, and the interaction parameter *U*^*R*^*n*_0_. Here, we included two phonon modes of frequencies Ω_*m*_*m*^0^ and $3{\Omega }_{m}^{0}$ (see Supplementary Note 8B for details). In the absence of interactions with the exciton reservoir (i.e., for ${U}_{n}^{R}0=0$), clear locking regions (shown in red) appear at even integer multiples of ${\Omega }_{m}^{0}$, as expected from the RWA. For increasing values of *U*^*R*^*n*_0_ odd integer multiples, as well as smaller fractional locking regions, also appear due to non-linear interaction effects (green regions).

So far, we have assumed that the traps are exposed to an externally generated phonon field. In reality, however, the phonon field is excited as part of the coherent locking process. Thus, a full self-consistent treatment requires to also solve, simultaneously to the polariton Eq. (1), the equation of motion for the mechanical displacement *x*_*m*_ (see Supplementary Note 8C). In ref. ^34^ it was shown that this model, even without interactions, leads upon continuous optical excitation to a parametric instability (similar to mechanical self-oscillation) that originates a large amplitude of the phonon field for certain (narrow) regions of detunings near $2{\Omega }_{m}^{0}$ and $4{\Omega }_{m}^{0}$. However, our model is an oversimplification of a very complex system, where different mechanism could in principle lead to some large fluctuation of the phonon field. In fact, the experimental results of ref. ^31^ suggest that this is the case as one observed intense phonon-induced PL satellite peaks. The latter is an indication of the presence of a large number of phonons in the system. Taking this into account, we solve the full equation self-consistently but introducing a large initial condition for *x*_*m*_ (specifically, *x*_*m*_ ~ Ω_*m*_/*g*_*m*_). This, together with the inclusion of the polariton non-linearities, leads to a much wider instabilities with asynchronous locking at all multiples of ${\Omega }_{m}^{0}$, as shown in Fig. 4g, where we present a histogram of $\Delta \widetilde{\omega }$ as done with the experimental data.

## Discussion

The polariton trap arrays studied here are of the same kind as the one investigated in ref. ^31^ in which polariton-driven phonon lasing was reported. The condition for the observation of such mechanical self-oscillation is that two polariton states have to satisfy the proper resonance condition, i.e., to be detuned by an integer number of phonon quanta, *n* > 1. The evidence for such phonon lasing was the observation of clear and intense sidebands separated by *ℏ*Ω_*m*_ from the emission of both ground and excited states of the traps. It follows from the extensive experiments reported here (see Supplementary Note 7) that phonon self-excitation does not necessarily lead to the appearance of sidebands. Sometimes we do indeed observe very clear and strong optomechanically-induced sidebands, in other cases we find that the levels asynchronously lock as reported here but there are no clear sidebands, and sometimes the two effects happen together (asynchronous locking plus weaker but discernible sidebands). Notably, from our systematical studies we find that the locking without intense sidebands is more frequent than the case of large amplitude sidebands. It seems also to be the case that a larger excitonic component favors the appearance of the sidebands. For the model in Eq. (2), inclusion in the rotating wave approximation of only the resonant terms leads to locking without sidebands. Consideration of the counter-rotating term is accompanied in the simulated spectra by sidebands. Moreover, it turns out that mechanical coherent oscillations of amplitude of just a few percent of the phonon energy are enough to establish the asynchronous locking, but would lead to very weak and, thus, hardly observable sidebands (see the Supplementary Note 8). It is our understanding that both, the emergence of sidebands and the asynchronous locking, are signatures of the existence of a mechanical coherent oscillation.

In summary, we have demonstrated a concept for polaromechanical metamaterials based on planar arrays of intra-cavity traps that confine, co-localize, and efficiently couple vibrations and polariton light fluids. The building blocks are micron-size high-Q resonators for polaritons and sound, that can be arranged and coupled in arbitrary tailored architectures. Polariton condensates ensure very long coherence times, exceeding the mechanical oscillation period, and lead through an exciton-mediated resonant interaction to hugely enhanced optomechanical couplings. Non-resonant continuous wave optical excitation results in mechanical self-oscillation and, through it, in the harmonic time modulation of the metamaterial parameters, with remarkable consequences for the coupled polariton and phonon dynamics. Non-linear interactions in this optomechanics setting contribute both to enhancing the optomechanical coupling,^29,47^ and to stabilize the locking. These results open the path for ultrafast GHz coherent mechanical control of light fluids in quantum technologies. Some examples are the use of phonon driving to induce coherent resonant inter-state transitions, for Floquet state engineering and Landau-Zener-Stückelberg state preparation^48^, and for the optomechanical induction of non-Hermitian chiral polaritonics^49^.

## Methods

### Photoluminescence spectroscopy

For the polariton PL experiments in the traps at 5-10 K, an external cavity-stabilized *cw* Spectra Physics Ti-Sapphire Matisse laser was used for the non-resonant excitation at ~ 760 nm. The wavevector-dependent energy dispersions in Fig. 2 were obtained by exciting the trap-arrays with a large ~ 50 μm laser spot, using small optical powers well below the condensation threshold. The wavevector dependence of the spectra was determined with standard methods based on angle-resolved light collection. The optical driving of the condensates to observe the reported asynchronous locking phenomena (Fig. 1c and 4) was performed through focused excitation purposely positioned in one of the central traps of the array using microcope optics to reduce the spot size down to ~ 3 μm. The same microscope objective (NA = 0.3) was used to collect the emitted light. In this latter case a triple additive Jobin-Yvon T64000 spectrometer was used to obtain the required high spectral resolution (~5 GHz^−1^–20 μeV).

### Pump and probe phonon spectroscopy

A ps-laser pulse is used to resonantly excite the optical cavity mode. A rapid change of index of refraction is induced by the pump through carrier excitation. In addition to this electronic response, the pump pulse launches coherent phonons by a displacive mechanism^50,51^. These mechanical oscillations modulate the cavity energy through two mechanisms, interface displacement and photoelastic interaction, which are detected using a delayed probe pulse that samples the cavity’s reflectivity. A typical spectrum displays characteristic lines corresponding to the ~ 20 GHz fundamental confined breathing mode of the structures, and weaker contributions at the higher energy overtones at ~60 GHz and ~100 GHz. More details are provided in Supplementary Note 2.

### Effective potential phonon modeling

We assume the non-etched effective quadratic dispersion relation arising when *k*_*z*_ is quantized, i.e., $E({k}_{x},{k}_{y})={E}_{{{{{{{{\rm{cav}}}}}}}},{{{{{{{\rm{ne}}}}}}}}}+{\hslash }^{2}({k}_{x}^{2}+{k}_{y}^{2})/(2{m}_{{{{{{{{\rm{eff}}}}}}}}})$, with homogenous in-plane speed of sound *v*_*s*_ defining an effective mass ${m}_{{{{{{{{\rm{eff}}}}}}}}}={E}_{{{{{{{{\rm{cav}}}}}}}},{{{{{{{\rm{ne}}}}}}}}}/{v}_{s}^{2}$. This is incorporated in a 2D Schroedinger-like equation,

3 $$\left[-\frac{{\hslash }^{2}}{2{m}_{{{{{{{{\rm{eff}}}}}}}}}}{\nabla }^{2}+{E}_{{{{{{{{\rm{cav}}}}}}}},{{{{{{{\rm{ne}}}}}}}}}+{V}_{e}(x,y)\right]\Psi (x,y)=E\Psi (x,y),$$

that adds the potential *V*_*e*_(*x*, *y*) to effectively describe the trapping induced by the etching. The full height of the potential in an etched region is ${V}_{\max }=({E}_{{{{{{{{\rm{cav}}}}}}}},{{{{{{{\rm{e}}}}}}}}}-{E}_{{{{{{{{\rm{cav}}}}}}}},{{{{{{{\rm{ne}}}}}}}}})$, with *E*_cav,e_ the energy of the phonon mode in a large etched region. Each square trap *i*, centered in (*x*_*i*_, *y*_*i*_) contributes to *V*_*e*_(*x*, *y*) the potential ${V}_{i}(x,y)={V}_{\max }[1-{v}_{i}(x-{x}_{i}){v}_{i}(y-{y}_{i})]$ where the trap profile along each direction is given by ${v}_{i}(\alpha )=\frac{1}{2}\left[{{{{{{{\rm{erfc}}}}}}}}\left(\frac{\alpha -\frac{{w}_{i}}{2}}{0.55{\delta }_{i}}\right)-{{{{{{{\rm{erfc}}}}}}}}\left(\frac{\alpha+\frac{{w}_{i}}{2}}{0.55{\delta }_{i}}\right)\right]$ with *w*_*i*_ the trap width and *δ*_*i*_ the 10% to 90% transition length. The eigenvalue problem is solved using finite differences by the customary approach of imposing periodic conditions fulfilling the Bloch theorem. For the width of the nER to ER transition regions, we take 0.35 μm, consistent with both the modeling of the polariton properties and STM studies in similar structures. More details and examples of the calculated phonon dispersion in trap arrays can be found in Supplementary Note 4.

### Estimation of the optomechanical coupling *g*_0_

Electrically generated mechanical waves have been used in individual similar traps to obtain *g*_om_/2*π* = Δ*E*_p_/Δ*u* ~ 50 THz/nm^28^ (change of polariton energy Δ*E*_p_ per unit displacement Δ*u*). This parameter is related to the on-site linear optomechanical coupling by *g*_0_ = *g*_om_*x*_zpf_ (*x*_zpf_ is the displacement due to zero point fluctuations)^18^. The effective mass associated to the oscillator can be estimated as *m*_eff_ ~ 0.5 pg for a structure of 2 μm lateral size (see the Supplementary Note 5) and from this we obtain *x*_zpf_ ~ 1 fm. It follows that *g*_0_/2*π* ~ 50 MHz, quite a huge value when compared with other reported optomechanical systems^18^. By involving the deformation potential interaction associated to the exciton component of polaritons, *g*_0_/2*π* is thus amplified by three orders of magnitude from the ~ 50kHz calculated for purely optical radiation pressure interaction^52^. To enhance cavity optomechanical phenomena materials with electronic resonances are usually avoided due to the related absorption that reduces the optical Q-factor^18^. Counter intuitively, the opposite occurs in our polaromechanical crystals. Indeed, the measured BEC coherence time is ~1 − 2 ns, implying *Q* ~ 7 × 10^5^, while the bare cavity photon lifetime is only ~10 ps. This means that, by involving the exciton-mediated optomechanical interaction, *g*_0_ is amplified by three orders of magnitude, while at the same time because the system is in the (polariton) strong coupling regime the Q-factor is enhanced by a factor ~100.

## Supplementary information

Supplementary Information

## Data Availability

The source data that support the findings of this study are available from the corresponding author upon request. All these data are directly shown in the corresponding figures without further processing.
