# Supplementary material for: Asynchronous locking in metamaterials of fluids of light and sound
Source: Nat Commun. 2023 Jun 19;14:3485. doi: 10.1038/s41467-023-38788-9 (PMC10279768; doi:10.1038/s41467-023-38788-9)
Supplement: Supplementary file 1 — Supplementary Information [file 41467_2023_38788_MOESM1_ESM.pdf]

Supplementary Material:
Asynchronous Locking in Metamaterials of Fluids of Light and Sound

D. L. Chafatinos,[1,2] A. S. Kuznetsov,[3] A. A. Reynoso,[1,2,4] G. Usaj,[1,2,5] P. Sesin,[1,2] I. Papuccio,[1,2] A. E. Bruchhausen,[1,2] K. Biermann,[3] P. V. Santos,[3,*] and A. Fainstein[1,2,†]

[1]*Centro Atómico Bariloche and Instituto Balseiro, Comisión Nacional de Energía Atómica (CNEA)- Universidad Nacional de Cuyo (UNCUYO), 8400 Bariloche, Argentina.*

[2]*Instituto de Nanociencia y Nanotecnología (INN-Bariloche), Consejo Nacional de Investigaciones Científicas y Técnicas (CONICET), Argentina.*

[3]*Paul-Drude-Institut für Festkörperelektronik, Leibniz-Institut im Forschungsverbund Berlin e.V., Hausvogteiplatz 5-7, 10117 Berlin, Germany.*

[4]*Departamento de Física Aplicada II, Universidad de Sevilla, E-41012 Sevilla, Spain*

[5]*TQC, Universiteit Antwerpen, Universiteitsplein 1, B-2610 Antwerpen, Belgium.*

(Dated: May 15, 2023)

SUPPLEMENTARY NOTE 1: SAMPLE DETAILS AND DESCRIPTION

A. The sample-fabrication

Figure 1(a) (top panel) shows a scheme of the profile of the planar sample. Here the real part of the refractive index corresponding to each layer is plotted as a function of the MBE growth direction (z). Figure 1(b) details the region of the hybrid photon/phonon cavity with the embedded QW's. The region of the cavity is highlighted in gray, and the region (ER) highlighted in green in the top panel of Fig.1(b) corresponds to the portion of the sample ($\sim$12 nm) that is removed during the etching process to attain the in-plane patterning required to define the traps [see Fig.1(a) of the main text].

The microcavities reported in this work were designed to optimize the *electrostrictive polariton-phonon interaction*. To achieve this situation, the quantum wells (QWs) were displaced from the anti-nodes of the cavity electric field [$E(z)$], which turn out to be nodes of the strain field [$s(z)$], to the position were the product $s(z)\ |E(z)|^2$ is maximized. The bottom panels of the respective Figs. 1(a) and 1(b) show this optimized magnitude along the z-direction for the planar sample. As can be noted, in panel (a) the strain-electric field product is peaked at the cavity spacer, and the overlap is optimal at the QWs position. This region of QWs and strain-electric field's superposition is indicated by the red-shaded area in Fig.1(b).

The fabrication method and micrometer size lateral patterning of the traps, in the context of cavity polariton, is well described in Refs.[1–3]. It consists of an MBE growth of the bottom DBR and the cavity spacer with the embedded QWs. The process continues with the patterning and wet-etching of the traps outside the MBE chamber, and is concluded with a subsequent MBE overgrowth of the top DBR. The sample consisted of a top(bottom) DBR formed by 25(33) $\frac{\lambda}{4}/\frac{\lambda}{4}$ periods of $Al_{0.15}Ga_{0.85}As/Al_{0.90}Ga_{0.10}As$ layers, embedding a $\frac{3}{2}\lambda$ cavity with four 15 nm GaAs QWs as schematized in Fig.1(b) (top panel), grown on a 350μm thick GaAs(001) substrate. A scheme of the resulting structure is shown in Fig.1(b) of the main text. One point worth mentioning is that, because the etching is performed at the limits of the cavity spacer, far from the QWs, the quality of the latter is not affected by the etching and overgrowth process.

B. Optimization of the electrostrictive polariton-phonon interaction

In Fig. 2 we schematically show how the values for the single-photon electrostrictive coupling rate g_0^{DP} (deformation potential interaction) evolves when the relative position within the cavity spacer is varied. As mentioned in the preceding paragraphs, and well observed in this figure, the polariton-phonon coupling strength is optimized when the quantum wells (QWs) are displaced in such way that the overlap between the product of $s(z)$, the acoustic strain field, and $|E(z)|^2$, the amplitude of the cavity's electric field, is maximized.

Figure 2(a) exemplifies the case of a simple λ cavity spacer (indicated by the gray-shaded area), for which the embedded single quantum well (SQW) is displaced from the cavity center. Superimposed (right y-axis), $|E(z)|^2$ and $s(z)$ are indicated by the full dark-green and dashed curves, respectively. Initially (violet curve, 0% off-centred), the SQW is centred at the electric field's anti-node, a situation that is usually chosen for enhancing the exciton-photon interaction. However, we notice that at that point the strain is zero so the electrostrictive polariton-phonon coupling vanishes. The SQW is gradually off-centred towards the front cavity spacer limit, departing from the optimal exciton-photon overlap but increasing its superposition with the acoustic confined strain field. Since g_0^{DP} is proportional to the overlap integral of $s(z)\ |E(z)|^2$ within the SQW [4], it will gradually change when the off-centring percentage is increased. In Fig. 2(b) we show $\int_{\mathrm{SQW}} s(z)\ |E(z)|^2\, dz$, where it is evident that the

* Corresponding author, e-mail: santos@pdi-berlin.de
† Corresponding author, e-mail: afains@cab.cnea.gov.ar

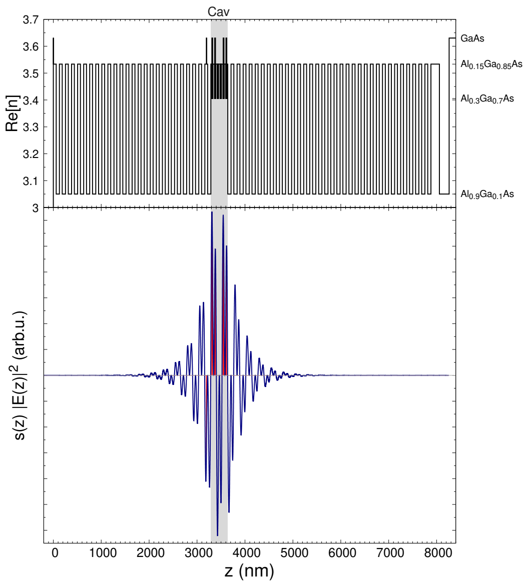

(a)

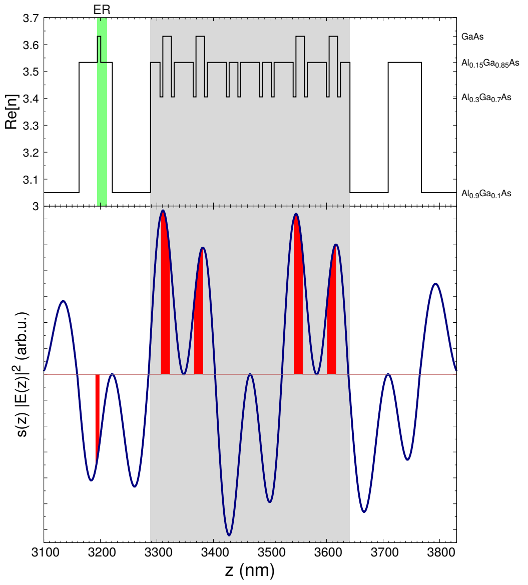

(b)

Supplementary Figure 1. **Sample's structure and strain-electric field overlap.** **(a)** The top panel displays the profile of the real part of the index of refraction (n) along the MBE growth direction z. z=0 nm corresponds to the air-sample surface, and the substrate begins at $\sim$8.2 μm. Top and bottom DBRs are separated by the cavity spacer (gray shaded region, Cav) that embeds the four GaAs QWs. The bottom panel shows the overlap between the strain field $s(z)$ and the squared electric field $E(z)$. **(b)** corresponds to the detail of the cavity region in **(a)**. The green area (ER) corresponds to the part that is removed during the etching process. The optimized overlap of $s(z)\,|E(z)|^2$ with the GaAs cavity QWs is indicated in red.

cavity centred QW is the worse situation. The optimal strain–e-field interaction, equivalently the optimal phonon-polariton interaction, is achieved at $\sim$11.5% off-centring [black thicker

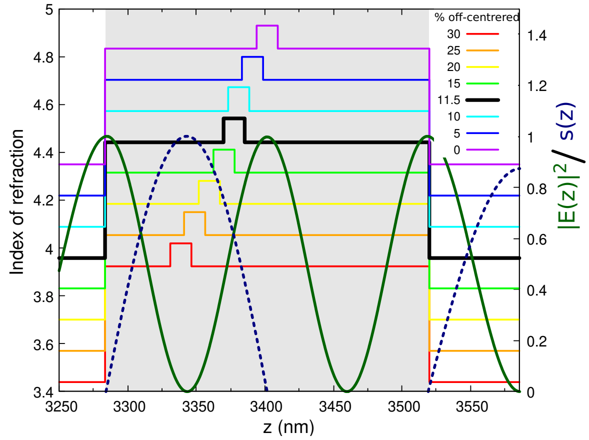

(a)

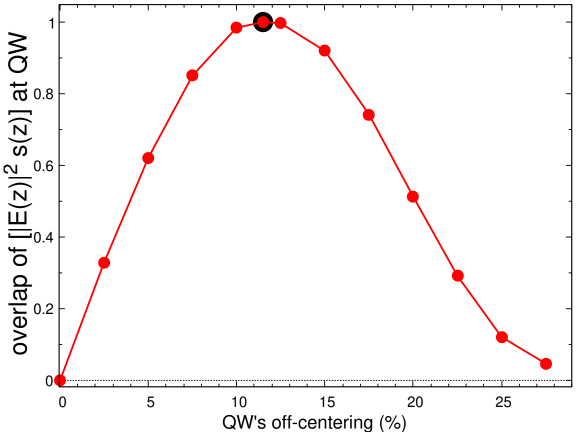

(b)

Supplementary Figure 2. **Optimization of the polariton-phonon coupling within a SQW cavity**. **(a)** Detail of a λ cavity spacer with an embedded SQW. The left y-axis corresponds to the index of refraction profile. Initially, the SQW is centered (0%) at the cavity spacer (gray-shaded region), and it is gradually off-centered. Superimposed (right y-axis), corresponds to the normalized electric field's intensity $|E(z)|^2$ (full dark-green) and acoustic strain $s(z)$ (dashed-blue). **(b)** Overlap integral of $|E(z)|^2$ and $s(z)$ for the different QW's positions depicted in panel (a). The optimized situation (maximum) is indicated by the black circle, which corresponds to the thicker black index of refraction profile in panel (a).

curve in Fig. 2(a)], and decreases afterwards.

C. List of the studied samples

In the Supplementary Table 1 we summarize the different samples studied in this work. The main characteristics that differentiate them are given: cavity-exciton detuning of non-etched region (Δ_{nER}) and the depth of the polaritonic barrier well (Δ_{LP}). The experimental technique used in each case is indicated: for phonon properties *Pump and Probe phonon spectroscopy* (P&P), and for polariton properties

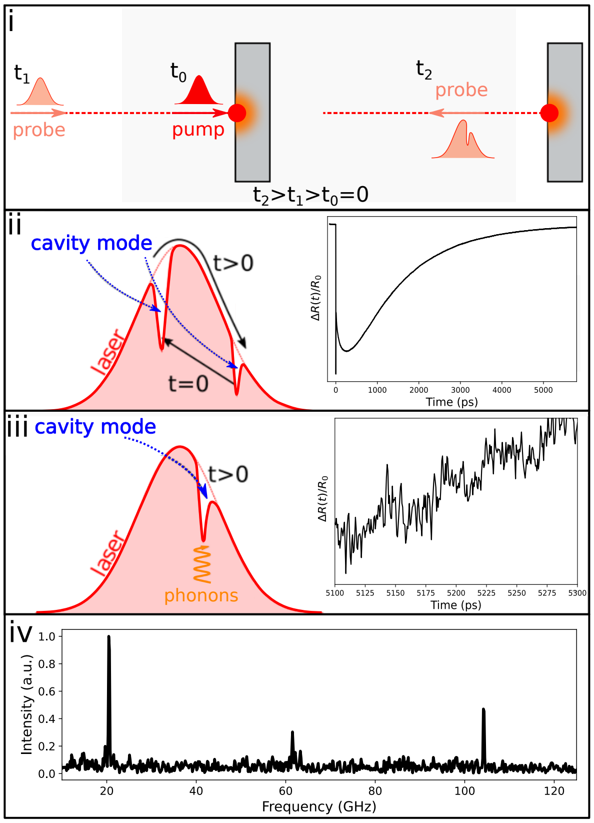

Supplementary Figure 3. **Picosecond coherent phonon pump-probe method.** A scheme of this technique is presented in panel (i). At t_0 the pump pulse strikes the sample inducing a change of the refractive index, and launching coherent phonons. At a later time t_1 a delayed pulse probes the cavity reflectivity. The change in the refractive index induces a shift of the cavity mode, leading to the so-called electronic signal (ii). The coherent phonons modulate the cavity mode leading to the vibrational signal (iii). Processing of the signal obtained as a function of the probe delay, and its Fourier transform, lead to the vibrational spectra (iv). Typical cavity confined modes are observed around 20, 60, and 100 GHz. Further details on the optical cavity mode dynamics and the impulsive coherent phonon generation and detection in microcavities and cavity-traps can be found in our Refs.[5] and [6].

Photoluminescence spectroscopy (PL). The figures of the main text and supplementary notes, where the corresponding data appears, are additionally included in column "Figure ID". Samples are either single traps, two coupled traps, or array of traps. They were designed and fabricated as explained above in this Supplementary Note 1.

SUPPLEMENTARY NOTE 2: TIME-RESOLVED PUMP-PROBE PHONON SPECTROSCOPY

The dynamical aspects of the phononic states of the individual traps, double-coupled traps, (molecules) and 2D trap arrays, were studied by ultra-fast laser spectroscopy using the picosecond coherent phonon pump and probe technique [7, 8]. In this work this technique was adapted for semiconductor optical microcavities [9], and combined with microscopy to enable the addressing of individual traps [10].

The concept of this method is illustrated in Fig. 3(i)-(iii). A ps-laser pulse is used to resonantly excite the optical cavity mode. Because of direct (above-gap excitation) or residual (below-gap excitation) carrier generation, a rapid change of index of refraction is induced by the pump, which recovers within a timescale defined by electron-hole evolution and recombination. As a consequence of this change in the refractive index, the cavity mode strongly blue-shifts in a ps scale, to recover its equilibrium in longer ns-scale times. In addition to this so-called electronic response, the pump pulse through the same carrier excitation launches coherent phonons mediated by a deformation-potential induced displacive mechanism [11, 12]. These mechanical oscillations modulate the cavity energy involving mainly two mechanisms, interface displacement and photoelastic interaction [11, 12], and can thus be detected using a delayed probe that samples the cavity's reflectivity [13, 14].

In Fig.3(ii)(right panel) a typically obtained transient reflectance $\Delta R(t)/R_o$ is shown. The reflectivity of the probe pulse is recorded as a function of the time delay with respect to the arrival of the exciting pump pulse that defines t=0 ps. Using a convenient filtering treatment, the slow varying envelope evolution, ascribed to the optical constant's temporal variations due to electronic states and thermal modifications, the faster dynamics defined by the vibrational oscillatory components can be extracted [see right panel in Fig3(iii)]. After extraction a spectral analysis is performed by applying a numerical Fourier transformation (nFT) of the measured window.
A typical spectrum is shown in Fig. 3(iv), with the characteristic stronger signal corresponding to the ∼20 GHz fundamental confined breathing mode of the structures, and weaker contributions at the higher energy overtones at ∼60 GHz and ∼100 GHz. These acoustic cavity modes have been previously studied in planar microcavities [9], and also in individual micrometer-size pillars obtained by deep etching planar structures [5, 15]. This previous knowledge is exploited here to experimentally identify the phonon confinement in *individual* polariton traps generated by weak distant modulation of the spacer thickness, and is extended to study more complex structures with different dimensionality (molecules, and 2D arrays).

SUPPLEMENTARY NOTE 3: PHONON CONFINEMENT IN TRAPS: EXPERIMENTAL RESULTS

Examples of measurements using this technique on a single micrometer size polariton trap are depicted in Fig.4. In panel (a), the transient curves corresponding to the vibrational contributions (the electronic part was removed as explained above), extracted for an isolated $2\,\mu\text{m}\times 2\,\mu\text{m}$ trap (pink curve), and for an isolated $10\,\mu\text{m}\times 10\,\mu\text{m}^2$ trap (violet curve) are shown. Both transients show clear oscillations, which

Type of sample	Structure parameters (μm)		Experimental technique	Δ_{nER} (meV)	Δ_{LP} (meV)	Figure ID
	Trap size	Separation				
Array of square traps	1.0	2.0	PL	-5.4	-7.1	4, S20, S21
Array of square traps	1.3	2.6	PL	-5.4	-7.1	4, 5, S16-S19, S21
Array of square traps	1.6	3.2	PL	-5.4	-7.1	1(c), S10-S15, S21
Array of square traps	1.0	1.0	PL	-10.6	-6.7	2(b)
Array of square traps	4.0	2.0, 1.0	P&P	-12.5	-10.8	3(d)
Array of square traps	2.0	2.0, 1.0, 0.5	P&P	-12.5	-10.8	3(d)
Double coupled traps	2.0	0.5	P&P	-12.5	-10.8	3(c)
Single square trap	10.0 - 1.0	–	P&P	-12.5	-10.8	3(b)
Single square trap	10.0 - 1.0	–	PL	-10.6	-6.7	3(a)

Supplementary Table 1. **Parameters of the different studied samples.** The first column describes the sample geometry. The second, the size and separation of the traps (except for isolated traps). The next column indicates the main experimental technique used in each experiment: Photoluminescence (PL) spectroscopy, and Pump and Probe (P&P) phonon spectroscopy. Δ_{nER} is the detuning between cavity-mode and exciton energies from the non-etched region (nER), and Δ_{LP} is the energy difference between the lower-polariton in the extended non-etched and etched regions. The latter defines the modulation of the polariton effective potential (as in Ref.[3]). The last column associates each sample with the data presented in the different figures of this work. The letter "S" describes a figure in the Supplementary Material.

result from the superposition of the spectral components [see Fig. 3(iv)] of ∼20, ∼60, ∼100 GHz (periods respectively of ∼50, ∼17, ∼10 ps). For the extended temporal scale chosen in Fig.4(a) the individual oscillations, which are coherent with respect the impulsive excitation of the pump pulse, cannot be distinguished. However, it is clearly noticeable that the signal remains basically undamped up to the observed ∼11 ns (the maximum scan time is limited by the ∼ 80 MHz repetition rate of the pulsed laser). It actually well exceeds this wide acquired time frame. To better see this prevalescence, in Fig.4(b) and (c) we show the transient oscillations of the fundamental mode of the traps, filtered using a band-pass ±3 GHz centered around the fundamental modes (at ∼20.68 GHz and ∼20.55 GHz, respectively). Figure 4(b) corresponds to a time window at early delay times, and Fig.4(c) at almost the end of the measured window. The observation of the vibrational modes of these micrometer size polariton traps, which exhibit lifetimes that well exceed 12 ns, is in contrast to what has been previously observed in optomechanical semiconductor micropillar resonators [5, 6, 16].

Several single square polariton traps of varying lateral sizes have been analyzed using picosecond coherent time-resolved pump-probe phonon spectroscopy in combination with microscopy. In Fig.5 we show a detail of the spectral region of the fundamental 3D confined phononic mode around 20 GHz [see Fig. 3(iv)], when varying the trap size as indicated. As a reference, the results for the planar samples on the non-edged (nER) and edged (ER) regions are included. Due to the measured temporal window (same for all traps, see Figs.4), the spectra are resolution limited. However, the blue-shift for decreasing trap size is clearly observed. The determination of the peaks' position, reported in Fig.3(b) of the main text, was performed by fitting the data using Gaussian distribution functions. The dotted vertical line indicates the frequency of the non-etched planar sample. The spectra for nER and ER have been measured using a shorter temporal window, with the consequent increase of the Fourier-limited peak's width.

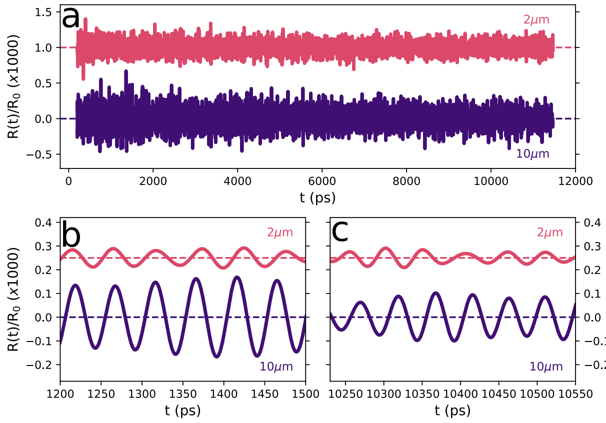

Supplementary Figure 4. **Extracted phonon oscillations for two different isolated traps.** **(a)** The bottom curve of this panel corresponds to the transient measured on a 10 μm × 10 μm trap, whereas the upper curve corresponds to the equivalent results on a 2 μm m× 2 μm trap. **(b)** and **(c)** correspond to the upper transients in **(a)** with a band-pass filter centered at 20.55 GHz and 20.68 GHz, respectively. **(b)** corresponds to a detail for short delay times, whereas **(c)** displays the situation near the end of the temporal measured window.

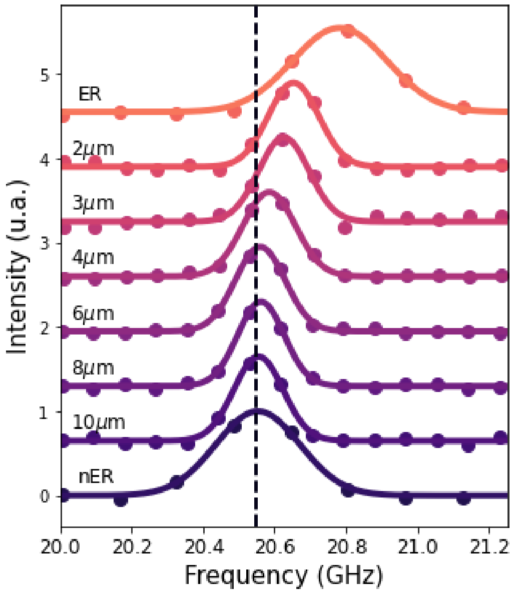

Supplementary Figure 5. **Variation with the traps' size of the confined fundamental phonon mode.** Spectra showing the size dependence of the acoustic confined ground state in square traps. Symbols are the experimental results obtained from a numerical Fourier transformation (nFT) of the extracted picosecond coherent time-resolved pump-probe measurements. The continuous curves are the corresponding Gaussian fits. The clear shift is evidenced, due to phonon confinement in the traps, with respect to the planar (non-structured) situation (nER) marked by the vertical dotted line.

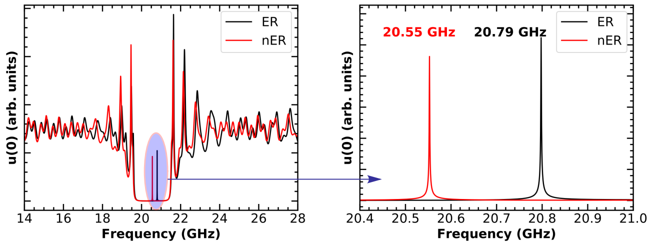

Supplementary Figure 6. **Phonon confinement in planar cavities.** The panel on the left shows the calculated surface displacement (equivalent to the transmission) for white sound propagating from the substrate for both the non-etched (nER) and etched (ER) planar regions. The calculations were performed using a transfer matrix method. The phonon cavity mode for each structure can be seen within the phonon DBR stop-bands (detailed with a zoom, right panel). The frequency difference (∼0.24 GHz) between both modes is a measure of the modulation amplitude for the effective phonon potentials affecting the in-plane propagation of these cavity-confined phonons when the lateral patterning defines the hybrid micrometer-size phonon-polariton traps.

SUPPLEMENTARY NOTE 4: THEORETICAL CONSIDERATIONS OF PHONON CONFINEMENT IN TRAPS AND ARRAYS OF TRAPS

A. Phonon confinement in planar structures

The vertical confinement (along the z direction) is illustrated in Fig. 6, where the calculated acoustic transmission is displayed for the cases of the planar non-etched (nER, red curve) and etched (ER, black curve) regions. The acoustic transmission is evaluated as the frequency resolved surface displacement for "white" sound incident from the substrate. The typical DBR phonon stop-band [17] can be identified between approximately 19.8 and 21.3 GHz.

The simultaneous photon-phonon field's co-localization of this hybrid device (see main text),[9] is evidenced for the non-etched region (nER) by the cavity mode appearing at the acoustic stop-band's center (∼20.55 GHz). The acoustic cavity mode for the etched region (ER) is blue-shifted out from the stop-band's center approximately by 0.24 GHz. Extending the same concept used for photonic traps to the acoustic phonons, one concludes that the lateral patterning described above should lead to an additional phononic lateral trapping potential, with modulations of ∼ 0.24 GHz between non-etched and etched regions. This potential should determine the in-plane propagation of the vertically confined acoustic phonons in a way that should be equivalent to the described confinement of photons [3].

B. 3D Phonon confinement: Finite elements calculation

To test and demonstrate the concept of lateral in-plane effective confinement in these kinds of traps, we calculated the mechanical eigenmodes using a commercial finite elements analysis software (COMSOL Multiphysics [18]) for the simplest geometry: a circular trap of cylindrical symmetry. GaAs and AlAs are considered as mechanically isotropic materials, and no mechanical absorption processes are considered. The used geometry is presented in Fig. 7(a). The material layering shown in this panel corresponds to the nominal values as defined by the MBE growth. The trap is modeled as a central circular region with larger spacer thickness as prescribed by the performed microfabrication process, and consistent with the measured polariton confinement (Fig. 1(a) of the main text). The full calculated structure is a cylindrical pillar limited by vacuum. In contrast with the optical response of the structure, phonons are subject to total reflection at the semiconductor/vacuum interfaces. To avoid any artificial effect induced by the pillar geometry, for the calculations we chose a total diameter large enough so that the free boundary condition of the sample-vacuum interfaces do not affect the solutions of modes localized at the smaller trap centered at the pillar. Also, in contrast with the light wave solutions, different mechanical directions of vibration are coupled through the Poisson's ratio (the ratio of transverse to axial elastic strain). In our case, a mode fulfills two simultaneous resonance conditions: the

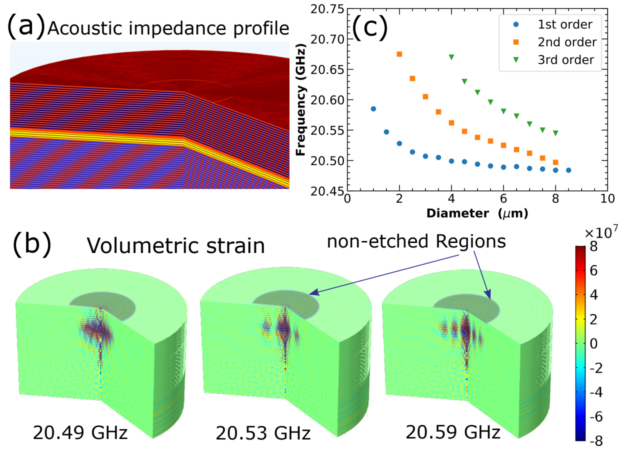

Supplementary Figure 7. **Phonon confinement in microcavity 3D traps.** Finite-elements 3D modeling of the spatial distribution and lateral size energy dependence, respectively, of the acoustic modes of single circular traps. **(a)** A detail of the modeled circular traps is presented, where the layered structure consists of: The top and bottom DBRs (indicated with red and violet layers, respectively), the spacer (yellow), and the patterned region (orange). The calculation is performed for a large cylindrical pillar of 20μm in diameter. At the center of this cylinder, the trap with the larger spacer thickness is patterned. The circular step forming the disk, is visible at the upper air-sample surface of this sketch (see text for details). In **(b)** the simulated volumetric strain ($\Delta v/V$) for first three confined mechanical modes is shown for a circular trap of a 5.5 μm diameter trap. **(c)** Results for the calculated frequency of the first three orders of mode confinement for corresponding varying trap diameter.

vertical confinement, determined by the DBRs and the spacer thickness, and the radial confinement determined by the effective potential induced by the local variation of the spacer thickness. Fulfilling both conditions results in a coupling between vertical and radial strains described by Poisson's ratio. Traps of diameter from 1-8 μm were modeled, choosing 20 μm for the external pillar diameter, and all the structure mounted on a 5 μm thick GaAs substrate.

As the material parameters, such as density, Young's modulus, and the Poisson ratio, well established and published values were used for the $Al_xGa_{1-x}As$ layered system [19]. The volumetric strain ($\Delta v/V$) for the mechanical fundamental eigenmode and the first two excited eigenmodes, for a 5.5μm diameter trap are shown in Fig. 7(b). These modes correspond to laterally well localized symmetric vibrations with different number of radial nodes. The trap-size dependence of the frequency of these modes is presented in Fig. 7(c), showing the expected $1/D$ increase with decreasing diameter D. For the ground state this confinement induced blue-shift amounts to somewhat less than 100 MHz for $D \sim 1\mu$m. Quite clearly the mechanical modes become strongly localized within the trap by the effective potential and, interestingly, due to the Poisson-ratio the breathing-like character of the modes implies that an expansion(compression) along z is accompanied by an in-plane compression(expansion).

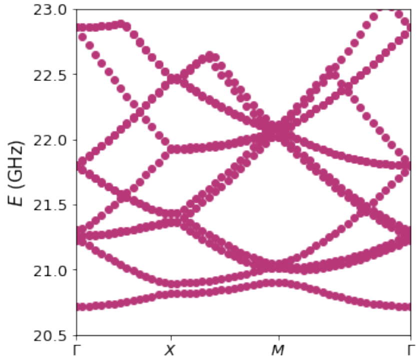

(a) 1 μm × 1 μm traps separated by 1 μm

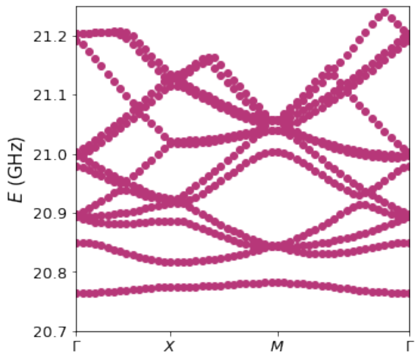

(b) 1 μm × 1 μm traps separated by 3.5 μm

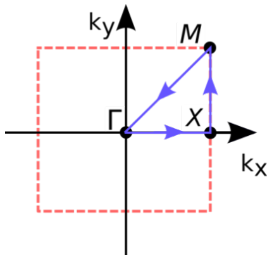

(c) Path followed in the 1st Brillouin zone

Supplementary Figure 8. **Phonons in polariton-phonon hybrid lattices.** The in-plane dispersion of the acoustic modes of a square lattice of 1 μm ×1μm square traps, separated by 1 and 3.5 μm, are shown respectively in **(a)** and **(b)**. **(c)** depicts the path followed in the first Broullin zone and shown in (a) and (b).

C. Effective potential model and phonon trap lattices

As addressed and demonstrated in the previous subsection A and B, the traps perform as resonators that confine phonons in all three dimensions. In addition, the finite height of the phonon effective potential barriers allows for the design of lattices, with the concomitant emergence of acoustic 2D phonon bands, in a similar way as what happens to polaritons.

To model how the cavity phonon mode's energy is affected by the different trap and array configurations (isolated traps, double-traps, and 2D arrays) we start from the non-etched effective quadratic dispersion relation arising when k_z is

quantized, i.e.

$$E(k_x, k_y) = E_{\text{cav,ne}} + \frac{\hbar^2(k_x^2 + k_y^2)}{2m_{\text{eff}}}. \quad (1)$$

Notice that for a material with homogeneous speed of sound v_s the effective mass $m_{\text{eff}} = E_{\text{cav,ne}}/v_s^2$. Such an effective mass description of the unconfined non-etched zone is incorporated in a 2D Schrödinger equation,

$$\left[-\frac{\hbar^2}{2m_{\text{eff}}}\nabla^2 + E_{\text{cav,ne}} + V_{\text{e}}(x, y)\right]\Psi(x, y) = E\,\Psi(x, y)\,, \quad (2)$$

that adds, via the potential $V_{\text{e}}(x, y)$, the effect of the trapping induced by the etching. Taking into account that the energy of the phonon mode in a large etched region is $E_{\text{cav,e}}$ we can assume that the full height of the potential in an etched zone is $V_{\max} = (E_{\text{cav,e}} - E_{\text{cav,ne}})$. Consequently, each square trap with a given index i, centered at (x_i, y_i) contributes to $V_{\text{e}}(x, y)$ the potential

$$V_i(x, y) = V_{\max}\left[1 - v_i(x - x_i)v_i(y - y_i)\right] \quad (3)$$

where, following Ref.[3], the profile of the trap "i" along each direction is given by

$$v_i(\alpha) = \frac{1}{2}\left[\text{erfc}\left(\frac{\alpha - \frac{w_i}{2}}{0.55\,\delta_i}\right) - \text{erfc}\left(\frac{\alpha + \frac{w_i}{2}}{0.55\,\delta_i}\right)\right], \quad (4)$$

where w_i is the trap width, δ_i is the 10% to 90% transition length, and erfc(...) stands for the *complementary error function*. Using finite differences we solve the eigenvalue problem by discretising a large 2D zone containing the traps. For the case of molecules and arrays, all traps have $w_i = w$ and are separated by the distance Δ. The simulated zone restricts to the supercell, where the super-lattice spacing is $a = w + \Delta$. After the numerical calculation, we obtain the eigen-energies for each value of $\boldsymbol{k}$ (1D or 2D) by the customary approach of imposing periodic conditions that fulfill the Bloch's theorem. For the width of the transition regions between the nER to ER, we take 0.35 μm, consistent with both the modeling of the polariton properties and scanning tunneling microscopy (STM) studies in similar structures[3].

The results of the calculations using the above described effective model are shown in Figs. 8 and 9. The acoustic phonon 2D band dispersion relation within the first Brillouin zone is shown in Fig. 8(a) for the case of a square array of 1 μm $\times$ 1 μm traps separated by 1 μm. The acoustic dispersion follows the usual path in k-space, i.e. $\Gamma \to X \to M \to \Gamma$, as indicated in panel (c) of this figure. Figure 8(b) corresponds to the acoustic phonon 2D band dispersion relation for the same square array of 1 μm $\times$ 1 μm traps, but with a distance between traps enlarged to 3.5 μm. As can be observed, only the lowest (fundamental) bands in (a) and (b) are energetically separated from the rest, resembling a tight-binding–like situation. The other higher energy bands are similar for both cases, resembling a free-electron–like physics. When analyzing the fundamental bands, it can be

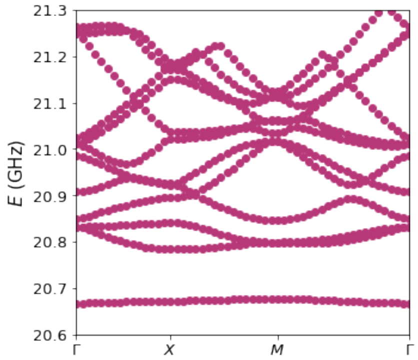

(a) 2 μm $\times$ 2 μm traps separated by 2 μm

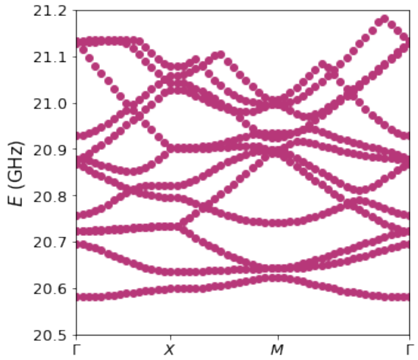

(b) Inverted
2 μm $\times$ 2 μm traps separated by 2 μm

Supplementary Figure 9. Phonons in polariton-phonon hybrid lattices: comparison with inverted potential lattices. (a) In-plane dispersion of the acoustic modes of a square lattice of 2 μm $\times$ 2 μm squared traps, separated by 2 μm. **(b)** Complementary lattice to (a) where the etched and non-etched regions are permuted.

noted that the case with more separated traps (3.5 μm barriers) displays a *flatter band*, that corresponds near the Γ point to more massive phonons [Fig. 8(b)]. Otherwise, the less separated traps (1 μm barriers) result in a broader band, and thus correspond to lighter phonons [Figs. 8(a)]. To be noticed here is that a pure 3D phonon gap around $\sim$ 20.75 GHz *only* exists for the thicker barrier [panel (b)].

The different bands result from the coupling of the discrete energy levels that are confined in 0D within each trap. For example, the lower energy band in Fig. 2(d) of the main text arises mainly from the fully confined s-like ground state of the phonon trap, and thus displays a tight-binding–like parabolic dependence around the Γ point, equivalent to the polariton case shown in panel (b) of that same figure. For this modelled structure, with small traps, the higher states are not confined and thus resemble those of almost free-electrons in a lattice.

A noticeable consequence is that the proposed technology can be used for phonon engineering in 2D based acoustic nanocavities, as has been previously proposed theoretically to obtain phonon molecules, band structures, and acoustic Bloch oscillations using simpler 1D layered media [20, 21].

Additionally, in Fig.9 two further examples of 2D dispersions for two trap arrays are shown. Panel (a) corresponds to a square array of $2\,\mu$m $\times$ $2\,\mu$m traps separated by $2\,\mu$m, and **(b)** for the inverted complementary structure, i.e. the regions that were etched and non-etched are inverted. The path followed to draw the 2D dispersion is the same as the one shown previously in Fig.8(c). In the first array (panel (a)) the phononic ground state is localized in the trap, with low overlap between traps. Contrarily, in the second array (the inverted one, panel (b)), the ground state is a more dispersive band.

D. Analogy between effective potentials for polaritons and phonons

The cavity exciton-polariton confinement in the growth direction (z) of the samples is determined by the photonic component. As explained in Ref.[9], for the $\mathrm{Al_xGa_{1-x}As}$–family based planar microcavity structures, due to a "magical coincidence" the optical and acoustic impedance mismatch is basically the same. Consequently, such a structure confining photons, identically confines phonons along the growth direction. Moreover, the confined photons and phonons share the very same wavelength, and their frequency difference is simply determined by the light-to-sound speed ratio. An important difference for phonons however is the existence of longitudinal acoustic (LA) and transverse acoustic (TA) modes. For planar systems grown in the [001]-direction, the above considerations apply for both acoustic polarisations separately. Because of the involved light-phonon coupling mechanism, in such planar structures only coupling to LA phonons becomes accessible.

Conceptually, photon trapped states (consequently polariton states) as those treated in this work, will have a z-component that resembles that of the planar structure, and additional in-plane components that will be defined by the propagation dynamics and lateral boundary conditions. Within a simple picture the same can be applied to phonons. The ground states of both will have similar wave-functions (both in-plane symmetric, 's'-like), and both will have in-plane anti-symmetric (p-type) first excited states. Similar lateral effective potentials are expected, *a priori*, given by the polariton or phonon frequency difference of the corresponding planar structure inside and outside the traps, an Ansatz that works well to describe our experimental findings.

The detailed character of the phonon modes in 0D traps, however, is more complex. When the in-plane symmetry is broken, as is the case of lateral micro-structuring for obtaining zero-dimensional (0D) trapped states, the acoustic vibrations are no longer pure LA and TA, but become mixed vibrational states, that -within a simple picture- are related by the Poisson ration. However, a detailed description of the corresponding mixed-vibrational eigen-states becomes non-trivial and numerical solutions need to be sought for [15, 16]. Although confined phonon states with similar envelope functions and spatial dependence to the case of the confined photons will exist, the rigorous quantitative description of the acoustic system will be more complex [15]. See Supplementary Note 4B for an example of such simulations.

Another important contrast between the phonon and polariton system, comes from the excitonic part of the polariton. The frequency of confined phonons and bare photons monotonically increases for decreasing cavity spacer thickness (i.e., for increasing amount of etching). However, this is not so straightforward in the polariton regime. Due to the exciton-photon strong coupling, the lower polariton level (from which the ground states of 0D traps and arrays are constituted) saturates at the exciton energy irrespective of the blue-shift of the bare photon states. Also the polariton effective mass depends on the cavity-exciton detuning. Consequently, the more excitonic the trap and barrier polaritons are, the weaker the analogy to the vibrational system is expected to be. In this work, the detuning is always kept negative for the trap states, i.e. the photonic components of the involved polariton states are dominant. But the energy of the barrier states is essentially saturated at the exciton energy. Qualitatively the analogy still holds, but one has to be careful when quantitative definitions are required.

In addition, the excitonic component of polaritons introduces another relevant difference when the particle occupation increased, due to the exciton-mediated polariton-reservoir and polariton-polariton interaction. For example, with increasing excitation power the confined polariton states blue-shift in an amount that is different for the non-etched well (more photonic) and etched barrier (more excitonic) regions of the effective polariton potential. These non-linearities do not exist for phonons.

SUPPLEMENTARY NOTE 5: CALCULATION OF THE EFFECTIVE MASS OF THE INVOLVED ACOUSTIC MODE

In order to obtain the effective mass (m_{eff}), a parameter that is usually used to describe the optomechanical coupling between the confined photonic (polaritonic) modes and a confined acoustic mode, we consider the involved potential energy. The general phonon displacement $\vec{U}(\vec{r})$ of the acoustic mode can be parameterized as $\vec{U}(\vec{r}) = u_0\,\vec{u}(\vec{r})$, where $\vec{u}(\vec{r})$ corresponds to the normalized displacement mode. The normalization of this mechanical mode is considered in such a way that at position $\vec{r}_0$, $|\vec{U}(\vec{r}_0)|^2 \equiv 1$. $\vec{r}_0$ is defined as the reduction point and is chosen as the position where the modes displacement is maximal.[25] For our system, the reduction point is situated at the interfaces of the phonon cavity spacer. The potential energy of the parameterized mechanical oscillator must be equal to the actual potential energy of the confined phonon breathing mode:

$$\tfrac{1}{2}\Omega_{\mathrm{M}}^2 \int d^3\vec{r}\,\rho(\vec{r})\,|\vec{U}(\vec{r})|^2 = \tfrac{1}{2}\,\Omega_{\mathrm{M}}^2\, m_{\mathrm{eff}}\, u_0^2\,. \quad (5)$$

Consequently, the effective mass is obtained as

$$m_{\mathrm{eff}} = \frac{\int d^3\vec{r}\,\rho(\vec{r})\,|\vec{U}(\vec{r})|^2}{u_0^2} \equiv \int d^3\vec{r}\,\rho(\vec{r})\,|\vec{u}(\vec{r})|^2\,, \quad (6)$$

where $\rho(\vec{r})$ is the density distribution field of the structure.

The parameterized effective equation of motion for the cavity confined breathing mode, corresponding to a damped driven oscillator, will be given by [26]

$$m_{\text{eff}}\frac{d^2u}{dt^2} + m_{\text{eff}}\,\Gamma_{\text{M}}\,\frac{du}{dt} + m_{\text{eff}}\Omega_{\text{M}}^2\,u = F\,. \quad (7)$$

Here m_{eff} is the effective mode's mass, and Γ_{M} represents the mechanical damping. The driving force F of the effective mechanical system involves the sum of different contributions, such as radiation pressure (F_{RP}) and electrostrictive (F_{ES}).

SUPPLEMENTARY NOTE 6: EFFECTIVE POLARITON TRAP POTENTIAL AND HIGHER-ORDER OPTOMECHANICAL COUPLING MECHANISM

A. The Gross-Pitaeskii equation with effective potential

The effective confinement potential for the polaritons is estimated using an effective Gross-Pitaeskii equation that takes into account both the blue shift induced by the repulsive interactions with the exciton reservoir and the saturation of the Rabi splitting [27]. The effective equation yields:

$$i\hbar\frac{\partial\psi}{\partial t} = \left[-\frac{\hbar^2\nabla^2}{2m_{\text{LP}}} + V_{\text{LP}}(\boldsymbol{r}) + \frac{i\hbar}{2}\left(\frac{R\,P(\boldsymbol{r})}{\gamma_{\text{R}} + R|\psi|^2} - \kappa\right)\right]\psi\,, \quad (8)$$

where $\psi(\boldsymbol{r},t)$ is the complex field describing the lower polaritons and we have used the adiabatic approximation for the exciton reservoir [28]. Equation (8) is a good approximation when describing the confined polaritonic levels s and p. In addition, in our particular case, and for the purposes of describing only the energy of the polaritonic modes (not their occupation) and the effective potential, this equation can be further simplified by ignoring the last term [27]. The effective potential is described as

$$V_{\text{LP}}(\boldsymbol{r}, n_{\text{R}}) = \frac{1}{2}\left[E_{\text{C}} + E_{\text{X}} - \sqrt{\Omega^2 + \Delta^2}\right]\,, \quad (9)$$

where $E_{\text{C}}(\boldsymbol{r}) = \Delta_0 + V_{\text{C}}(\boldsymbol{r})$ describes the photonic potential of the trap, Δ_0 the bare detuning, $E_{\text{X}}(\boldsymbol{r}, n_{\text{R}}) = g_{\text{X}}n_{\text{R}}(\boldsymbol{r})$ the exciton energy ($g_{\text{X}} \approx 6\,\mu\text{eV}\mu\text{m}^2$) and $\Delta(\boldsymbol{r}, n_{\text{R}}) = E_{\text{C}}(\boldsymbol{r}) - E_{\text{X}}(\boldsymbol{r}, n_{\text{R}})$ the effective detuning. Here we have ignored the contribution to E_{X} from the repulsive interaction among the lower polaritons. This is fine in this case as the polaritons have a large photonic component.

The saturation of the Rabi splitting with increasing population of the reservoir is described as

$$\Omega(\boldsymbol{r}, n_{\text{R}}) = \frac{\Omega_0}{\sqrt{1 + \frac{n_{\text{R}}(\boldsymbol{r})}{n_{\text{Sat}}}}}, \quad (10)$$

where Ω_0 is the Rabi splitting at zero carrier density and $n_{\text{Sat}} \approx 3\times10^3\mu\text{m}^{-2}$. The effective mass is approximated as

$$\frac{1}{m_{\text{LP}}} = \frac{|X(\boldsymbol{r}_0, n_{\text{R}})|^2}{m_{\text{X}}} + \frac{|C(\boldsymbol{r}_0, n_R)|^2}{m_{\text{C}}}\,, \quad (11)$$

where $\boldsymbol{r}_0$ corresponds to the center of the pumped trap and the spatially dependent Hopfield coefficients are

$$|X(\boldsymbol{r}, n_{\text{R}})|^2 = \frac{1}{2}\left(1 + \frac{\Delta(\boldsymbol{r}, n_{\text{R}})}{\sqrt{\Omega(\boldsymbol{r}, n_{\text{R}})^2 + \Delta(\boldsymbol{r}, n_{\text{R}})^2}}\right)\,,$$
$$|C(\boldsymbol{r}, n_{\text{R}})|^2 = 1 - |X(\boldsymbol{r}, n_{\text{R}})|^2\,. \quad (12)$$

Note that we have explicitly taken into account the dependence of the parameters with the density of carriers in the reservoir. The density of excitons in the reservoir as a function of the external pump power is estimated as

$$n_{\text{R}}(\boldsymbol{r}) = \frac{P(\boldsymbol{r})\tau_{\text{R}}\alpha}{\hbar\omega_{\text{L}}\,2N_{\text{QW}}}\,, \quad (13)$$

where $P(\boldsymbol{r})$ is the pump power per unit area, τ_{R} is the effective lifetime of the exciton in the reservoir, α is the total effective absorption coefficient of the QWs, $\hbar\omega_{\text{L}}$ is the energy of the non-resonant pumping laser, N_{QW} is the number of quantum wells and the factor 2 accounts for the dominant role of the triplet interactions. We assume a Gaussian shape for the pump given by

$$P(\boldsymbol{r}) = \frac{P_0}{2\pi\sigma_{\text{p}}^2}\exp\left[-\frac{(\boldsymbol{r}-\boldsymbol{r}_{\text{p}})^2}{2\sigma_{\text{p}}^2}\right] \quad (14)$$

In the simulations we use an effective value for the standard deviation $\sigma_{\text{p}} \approx 3\mu$m, and change the position of the spot ($\boldsymbol{r}_{\text{p}}$) to reproduce the particular experimental situation. The values for Ω_0, Δ_0 and the cavity parameters m_{C} and $V_{\text{C}}(\boldsymbol{r})$ were obtained by fitting. The photonic cavity potential $V_{\text{C}}(\boldsymbol{r})$ was simulated following Refs. [3]. The optimal results were obtained using $\Omega_0 = 6.0$meV and $E_{\text{C}} = \Delta_0 = -10.5$ meV and $E_{\text{C}} = \Delta_0 + \Delta U_{\text{p}} = 5.5$ meV for the non etched and etched regions, respectively. Here $\Delta U_{\text{p}} = 16$ meV is the potential barrier for photons, generated by the difference in the thickness of the cavity spacer between the two regions.

According to the model described above, in Fig. 10 we show the simulated effective trap polariton potential profile for two 1.6μm×1.6μm square traps separated by 3.2μm, and two different non-resonant pump-power conditions, i.e. low- (left panel) and high-power (right panel) excitation. This modelling corresponds to the studied case of traps of the largest inter-trap separation, with experiments shown in Figs. 1 and 4 of the main text. On each panel, the corresponding confined polariton wave-functions for each trap, derived from the Gross-Pitaevskii equation, are shown superimposed. The detuning between the pumped and neighbor trap ground states can be tuned through the excitonic-related repulsive interaction with the reservoir by varying the non-resonant pump-power. Notice that the polariton levels within each trap shift rigidly for increasing

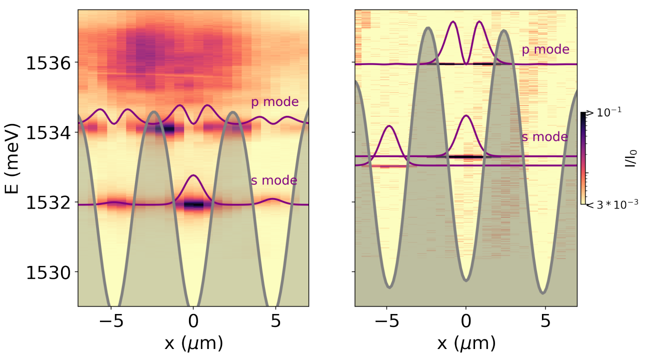

Supplementary Figure 10. **Effective polariton trap potential for two** 1.6μ**m**$\times1.6\mu$**m square traps separated by** 3.2μ**m.** Panel left (right) displays the spectral and spatial image corresponding to the low (high)-pump power condition. The effective trap potentials (shaded gray) that result from a Gross-Pitaevskii (GP) modelling of the quantum light fluid. Two situations are shown, corresponding to a power excitation condition below (left panel) and above (right panel) the condensation threshold P_{th}. The corresponding GP calculated confined polariton wavefunctions (thin purple curves) of the fundamental 's'-type and first excited 'p'-type modes are also plotted and indicated. The underlying density plot corresponds to the experimental measured photoluminescence at the corresponding excitation power.

power. As observed, in Fig. **10**, the 's'-type confined ground states are at near zero detuning at low power (left panel), while the detuning is evidently increased at high power (right panel).

We would like to point out, that the overlap between the 's'-type confined ground states of the central trap and the neighboring trap is very small ($\sim 10^{-4}$). These states turn out to be strongly confined within each trap, resulting in a negligible $s-s$ transition probability, and consequently a negligible inter-trap optomechanical coupling.

On the contrary, as observed in Fig. **10**, the penetration into the barriers of the first excited ('p'-type) confined states is significantly higher than that of the 's' states. The extension and "delocalization" of these excited states is clearly evidenced. For these confined 'p' states, the simulation shows that the overlap between neighbor traps' wave-functions is ~0.4, implying a much larger transition probability as compared to the s–s case [27]. The underlying density-plot corresponds to the system's experimental photoluminescence, where the low- (left panel) and high-power (right panel) situations are very well described by the model.

In this array of rather distant traps, the negligible overlap between ground states of neighbor traps, and the delocalization of the first excited states, justifies a Hamiltonian model for the inter-trap optomechanical coupling that considers a second order process mediated through the p-states to couple the two fundamental s modes.

B. Second order optomechanical coupling rate g_2

The negligible inter-trap optomechanical coupling of the traps' ground states in arrays of relatively distant traps (see previous subsection), and the experimental evidence of the strong interconnection between the neighbour traps in the 1.6μm$\times1.6\mu$m square trap array, which lead to the appearance of induced sidebands on the photoluminescence (PL) spectrum [10] at phonon energy separations, drives us to introduce a simplified model with a *second order inter-trap optomechanical coupling* mechanism, that indeed captures the main physical ingredients [27].

The model takes into account the two fundamental polaritonic modes of two neighboring cavities, a single polariton excited state that is shared by *both* traps, and an on-site phonon-mediated coupling between ground and excited states. The Hamiltonian then has two contributions, $H = H_0 + H_{\rm OM}$. Here

$$H_0 = \sum_{j=1}^{3} \hbar\omega_j\, \hat{a}_j^\dagger \hat{a}_j + \sum_n \hbar\Omega_n\, \hat{b}_n^\dagger \hat{b}_n\,, \qquad (15)$$

describes the decoupled polariton and phonon modes: i) $\hat{a}_j^\dagger$ ($\hat{a}_j$) creates (annihilates) a polariton in the j-mode with energy $\hbar\omega_j$, where $j=3$ refers to the excited mode; ii) $\hat{b}_n^\dagger$ ($\hat{b}_n$) creates (annihilates) a p-phonon in the n-mode with energy $\hbar\Omega_n$. The index n labels the fundamental and the overtone mechanical modes so that, for example, $\Omega_1 = 3\Omega_0 \sim 2\pi\times$ 60GHz (for simplicity, we take $\Omega_0 = 2\pi\times20$GHz). The linear optomechanical coupling reads

$$H_{\rm OM} \;=\; \sum_{j=1}^{2}\sum_n \hbar g_{jn}(\hat{a}_j^\dagger\hat{a}_3 + \hat{a}_3^\dagger\hat{a}_j)(\hat{b}_n^\dagger + \hat{b}_n)\,. \qquad (16)$$

The optomechanical features in the PL spectrum appear when the modes $\hat{a}_1$ and $\hat{a}_2$ are tuned to a particular energy difference (related to the phonon frequencies). This calls for a description where these two modes play the more important role. Since the excited mode is well-separated from the fundamental modes in comparison with the phonon energy, $\Delta_j = \omega_3 - \omega_j \gg \Omega_n$, it is thus reasonable to assume that one can describe the dynamics with an effective reduced Hamiltonian. This can be done by means of a suitable canonical transformation [27]. It can be readily seen that, to leading order in g_{jn}/Δ_j and Ω_n/Δ_j, and retaining only those terms involving the phonon operators,

$$\begin{aligned} H' \;=\; & \sum_{j=1}^{3} \hbar\omega_j\, \hat{a}_j^\dagger\hat{a}_j + \sum_n \hbar\Omega_n\, \hat{b}_n^\dagger\hat{b}_n \\ & + \sum_{j=1}^{2}\sum_{n,m} \frac{\hbar g_{jn} g_{jm}}{\Delta}(\hat{a}_3^\dagger\hat{a}_3 - \hat{a}_j^\dagger\hat{a}_j)(\hat{b}_n^\dagger + \hat{b}_n)(\hat{b}_m^\dagger + \hat{b}_m) \\ & - \sum_{n,m} \frac{\hbar g_{1n} g_{2m}}{\Delta}(\hat{a}_1^\dagger\hat{a}_2 + \hat{a}_2^\dagger\hat{a}_1)(\hat{b}_n^\dagger + \hat{b}_n)(\hat{b}_m^\dagger + \hat{b}_m)\,. \end{aligned} \qquad (17)$$

Here, for simplicity, we took $\Delta_j \pm \Omega_n \sim \Delta_j \equiv \Delta$. The second line in H' reflects a coupling between the phonon

displacement and the polariton mode occupations. This leads to a renormalization of the phonon energies induced by the polaritons in both the ground and excited states. When a mechanical coherent state sets in, it also leads to a modification of the polariton energies depending on the phonon occupation. The last line in H', in turns, makes explicit that there is an effective *quadratic* phonon coupling between the two fundamental polariton modes of the neighboring traps, of order $g_2 = g_0^2/\Delta$ [27].

C. Estimation of the on-site and the inter-site optomechanical coupling rate

The linear on-site optomechanical coupling factor g_0, which is governed by the processes that couple two polariton levels within the *same* trap, is estimated considering the effective exciton-mediated optomechanical coupling reported for similar polariton traps as those considered in this work [22].

The on-site coupling in Ref. [22] results mainly from a deformation-potential interaction modulated by intense electrically generated bulk acoustic waves (BAWs). A value of $g_{\rm om}/2\pi \sim 50$ THz/nm was experimentally obtained, which accounts for the change of polariton energy per unit of acoustic displacement.

This later parameter is related to the actual on-site optomechanical coupling constant (g_0) considering the displacement induced by the zero point fluctuations ($x_{\rm zpf}$) by $g_0 = g_{\rm om}\, x_{\rm zpf}$ [23]. For a similar structure of $\sim 1 - 2\,\mu$m lateral size, this value has been estimated to be roughly $x_{\rm zpf} \sim 1\,$fm [24]. Consequently, $g_0/2\pi \sim 50$ MHz for this system, which represents a very large value compared to other reported optomechanical systems that only account for an optical radiation back-action mechanism based on radiation pressure interaction [23]. The Hopfield coefficient for this cavity polariton trap system was of the order of $|X|^2 \sim 0.7$, and the reported structure had only one embedded QW [22].

The structure investigated in this work, has four cavity embedded QWs instead of one, proportionally increasing the corresponding interaction of the involved fields. For high excitation powers used for exciting the traps analyzed here, the excitonic Hopfield coefficient is estimated to be around $|X|^2 \sim 0.05$, i.e. the involved polariton states have larger photonic components. Considering these two differences, the actual g_0 would be roughly a factor of 4 times larger and a factor 0.05/0.7 smaller with respect to the above obtained value. Therefore, the on-site optomechanical coupling factor for the present work, resulting from a deformation-potential interaction, can be estimated to be $g_0/2\pi \sim 14\,$MHz.

Based on this estimated on-site (intra-trap) optomechanical coupling factor, considering the model discussed in the previous subsection, and accounting that the energy distance between the excited state and the ground state is $\Delta_j \approx 2\,$meV (∼484 GHz) (see e.g. Fig.**10**), we can estimate an inter-trap single-photon coupling rate of $g_2 = g_0^2/\Delta \sim 400\,$Hz. This inter-site second order coupling was shown in Ref.[27] to lead to the development of mechanical self-oscillation due to an optomechanical parametric process in the resonant condition when polaritons condense. That is, if the energy separation between neighbor traps is 2Ω, in Ref.[10], the magnitude of the polariton energy modulation induced by this coherent self-substained mechanical wave was estimated to be a quite large fraction ∼0.6 of the mechanical energy $\hbar\Omega$.

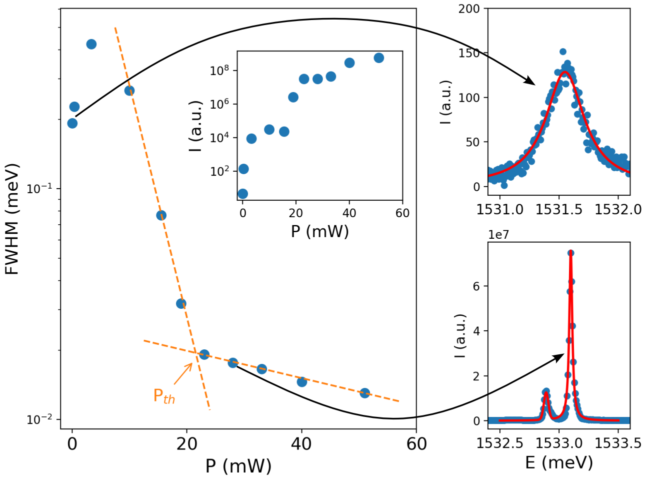

Supplementary Figure 11. Determination of the condensation threshold power $P_{\rm th}$. Left panel: pump power dependence of the full width at half maximum (FWHM) of PL spectra of the polaritonic fundamental mode of the pumped trap (in logarithmic scale). The condensation threshold power $P_{\rm th}$ is identified by the intersection of the change in slope of the FWHM as a function of power (orange dashed lines). The inset panel shows the intensity of fundamental pumped mode (in logarithmic scale). The right panels show the measured PL spectra (blue dots) for two pump-power situations, indicated correspondingly by the black-arrows. The top(bottom) right panel depicts the situation before(after) condensation. For the latter two fundamental modes can be identified: the one of the central pumped trap (at ∼1533.1 meV), and a neighbor trap to lower energy.

For a driven cavity the single polariton g_0 is amplified as $g_{\rm eff} = g_0\sqrt{N_{\rm p}}$, [23] with $N_{\rm p}$ the polariton occupation ($N_{\rm p} \sim 10^6$ in the condensed phase [10]). Consequently, $g_{\rm eff}/2\pi \sim 14$ GHz. This value brings the system potentially into the so-called ultra-strong-coupling regime, where the polariton-phonon coupling rate becomes much larger than the phonon and polariton decay rates ($\Gamma_{\rm m} \sim 50$ MHz and $\kappa \sim 1$ GHz), but also of the order of the phonon frequency ($\omega_{\rm m}/2\pi \sim 20$ GHz).

SUPPLEMENTARY NOTE 7: EXPERIMENTAL RESULTS OF POLAROMECHANICAL PROPERTIES

A. Condensation threshold

The pump power threshold of the polariton condensation $P_{\rm th}$ was established by analysing the photoluminescence (PL) of the fundamental polaritonic mode of the pumped central trap. For determining $P_{\rm th}$, the full width at half maximum

(FWHM) was extracted as a function of excitation power, as shown in Fig. **11** (left panel). For $P \ll P_{\mathrm{th}}$, with increasing pump power, the polaritonic mode broadens due to an increase in polariton decoherence, consequence of the polaritonic self-interaction noise introduced by polariton-polariton and polariton-reservoir interactions. Afterwards, the mode's width drops off steeply evidencing the transition to the condensed phase [29]. For $P \sim P_{\mathrm{th}}$, the PL line-width changes its evolving slope (as indicated in Fig. **11**). P_{th} is determined as the power for which this change is noted (see orange dashed lines). The inset of the left panel shows the PL intensity as a function of excitation power. A change in the evolution of this magnitude is observed near $P \sim P_{\mathrm{th}}$. In the right panels of Fig. **11**, typical spectra of the fundamental modes' PL, before (top-right) and after (bottom-right) condensation, are shown. The increase of five orders of magnitude in intensity signals the transition to the condensed phase (see inset).

B. Mechanical modulation of polariton modes: Appearance of side-bands

As we have reported in Ref.[10], an important characteristics that is observed when two involved polariton states (the fundamental modes of the central and neighbour traps in this case) satisfy a proper resonance condition $\delta\nu = \nu - \nu_0 = -2 \times n \times \nu_m$ (with $n > 0$ an integer number and ν_m the fundamental mechanical mode), is the appearance of strong side-bands. The emergence of symmetrically and equally spaced side-bands, evidences that the system has entered a condition of mechanical coherent self-oscillation. Indeed, it has been shown that this phenomenon corresponds to a distinct type of optomechanical parametric oscillation [27], arising from an inter-site optomechanical coupling that is quadratic in the mechanical displacement. In Fig. **12**(a), the orange spectrum corresponds to a near-resonance situation $\delta\nu \approx -2\nu_m$. Here, clear side-bands are observed (white arrows) for the central trap at $\nu - \nu_0 = 0$. However, as mentioned in the main text, it turns out that this is not the most general case. In fact, in many cases we have observed asynchronous locking of polariton trap modes at multiples of the phonon frequency (something that we conclude is evidence of the existence of a self-induced coherent mechanical wave), *without* clear side-bands being observed.

As an example taken from the data originally published in Ref.[10], we present the blue spectrum in Fig. **12**(a) corresponding to the situation were the resonance condition is satisfied, $\delta\nu = -2\nu_m$. In this case, notably a third trap's fundamental mode suggestively also appears at $\delta\nu = -4\nu_m$. However, no evidence of side-bands is found. Another example of such asynchronous lockin3.2μm. From bottom to top the evolution of the PL of the traps' modes is plotted for increasing power. It is observed that, above the condensation threshold, four trap modes appear. At the highest pump power, all modes seem to lock asynchronously at a separation of $2 \times \nu_m$ with each other. However, again clear symmetrical side-bands were not observed. As we argue in the main text, and in Supplementary Note 8B below, this can be understood as related to the amplitude of the self-induced mechanical perturbation: much smaller mechanical amplitudes are required to induce the locking, than those needed to generate experimentally observable PL side-bands.

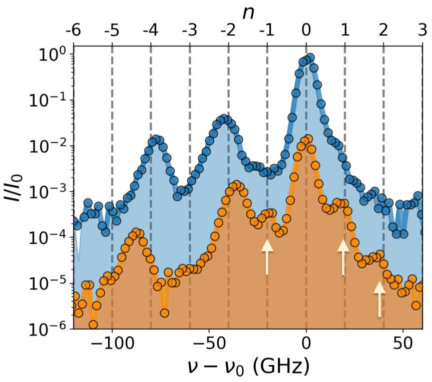

(a)

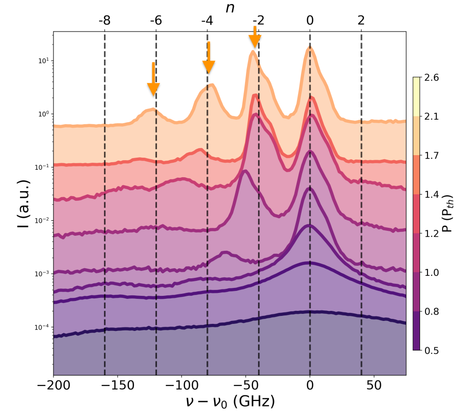

(b)

Supplementary Figure 12. **Appearance of sidebands by optomechanical interaction.** **(a)** PL emission with (orange spectrum) and without (blue spectrum) side-bands (indicated with white arrows at multiples of $\nu_m \sim$ 20GHz). Here the frequency scale is given relative to the energy of the fundamental mode of the central pumped trap. The fundamental mode of the nearest neighbor trap can be observed at $\nu - \nu_0 \sim -2\nu_m = -40$GHz. A third fundamental mode appears at lower relative frequencies for both cases, corresponding to an additional neighbor trap. Furthermore, for the blue spectrum, this third mode also tunes at an integer multiple of phonon frequencies, $\nu - \nu_0 \sim -4\nu_m = -80$GHz. This data has been adapted from Ref. [10]. **(b)** Cascade of spectra for varying excitation power (increasing from bottom to top) with light focused on another trap of the same array of 1.6μm×1.6μm square traps, separated by 3.2μm. Note that above the pump power condensation threshold, four clear modes appear (indicated with orange arrows), which suggestively appear at multiples of $2 \times \nu_m$ for the highest powers. In this case the mode locking occurs *without* the presence of sidebands.

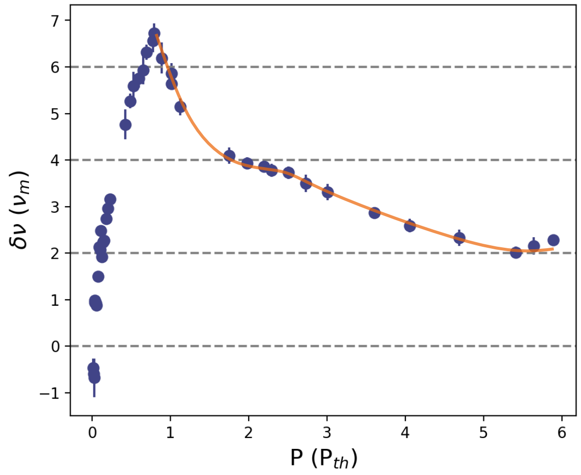

Supplementary Figure 13. Preliminary evidence of asynchronous locking. Frequency detuning (given in terms of fundamental mechanical frequency ν_m) between the pumped and the neighbor trap as a function of pump power (given in terms of $P_{\rm th}$, the condensation threshold power). The red full line is a guide to the eye. The data corresponds to Ref.[27].

C. Further examples of asynchronous locking of the polariton modes

While Ref.[10] reported the observation of mechanical self-oscillation when the proper resonant detuning between the fundamental modes of neighbor traps was satisfied, at that stage it was not yet realized the existence of the asynchronous locking reported here. Nevertheless, when the data presented in Ref.[10] is reanalyzed, it is evidenced that signatures of this phenomenon were already present in those experiments. Figure 13 shows, for that original set of data, the frequency detuning (given in terms of the fundamental mechanical frequency ν_m) between the pumped and the neighbor trap as a function of pump power (given in terms of $P_{\rm th}$, the condensation threshold power. The horizontal dashed lines indicate the matching of this detuning with even multiples of ν_m. Because one trap of the array is preferentially pumped, the detuning initially strongly increases due to interactions that are larger in the pumped trap, up to a point of saturation. On further increasing the excitation power, above $P_{\rm th}$ the detuning between modes diminishes when the neighbor trap (and its reservoir) increases their occupation. Quite notably, when $\delta\nu$ reaches a value near $4\nu_m$ the evolution gets into a plateau (partial locking). When further increasing the excitation power, the detuning continues to reduce, and finally decreases again its slope and stabilizes at a separation of $2\nu_m$ for the highest attained powers. We conclude that, in retrospective, the experiments in Ref.[10] also provide preliminary evidence of the asynchronous locking reported here. In what follows we extend on a comprehensive study of a series of arrays of traps of different sizes and inter-trap separation that, together with the data in the main text, provide the conclusive experimental evidence of the novel polariton-phonon synchronization phenomena.

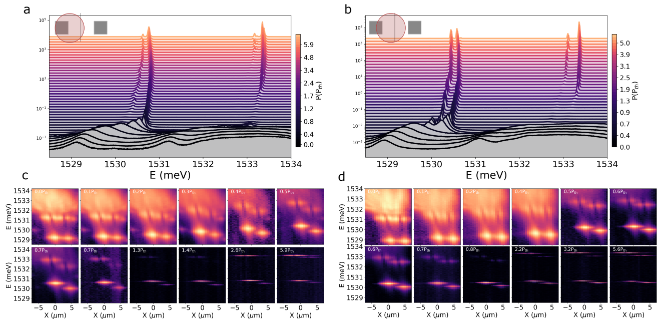

Supplementary Figure 14. Raw PL data from the array of 1.6μm$\times$$1.6\mu$m square traps separated by 3.2μm (a) and **(b)** present cascades of spectra for increasing excitation power (from bottom to top), for the excitation laser focused on two different positions in between two succesive traps of the array (indicated in the top schemes). **(c)** and **(d)** are the corresponding spectrally resolved spatial images. These raw data correspond to the results presented in Fig.4 of the main text.

D. Experiments on an array of 1.6μm$\times$$1.6\mu$m square traps separated by 3.2μm

Figure 14 illustrates two series of photoluminescence experiments performed at 10 K in an array of 1.6μm$\times$$1.6\mu$m square traps separated by 3.2μm. This array, as discussed in Supplementary Note 6, corresponds to the case of rather isolated traps, in which the inter-trap coupling is mediated by the mechanics leading to an optomechanical interaction that is quadratic in the phonon displacement. The top Figs 14a and b present spectra for different excitation powers as a cascade with power increasing from bottom to top, for two different spot positions in between two traps of the array (these are the kind of raw data from which Fig.4 of the main text is derived). The corresponding spatial images, that allow for an identification of the origin of each observed spectral peak, are presented in Figs 14c and d. In these experiments a full filling of the microscope objective pupil was performed so as to attain the smallest possible laser spot size (around 3μm). This, together with an improved spot positioning, allowed to perform experiments in which individual traps are better addressed, and the spot position could be finely controlled on top or at varying positions in between successive traps of the array (as indicated in the schemes in the top of Fig. 14). These experiments show the expected fundamental (s-like) and excited (p-like) states of neighbor traps of the array. They also show how the corresponding peaks blue-shift and narrow with increasing power, evidencing the Coulomb repulsive interaction and the passage to condensation, respectively.

In the spectra in Fig. 14, due to the set experimental conditions, two neighbor traps mainly contribute to the emitted light. From the spectra it follows that the energy detuning between the two involved ground states decreases with increasing power, keeping a finite separation even at the highest attained powers (around 6 times the condensation threshold power). The power dependence of this frequency detuning is shown in Figs. 15a-e, for five different positions

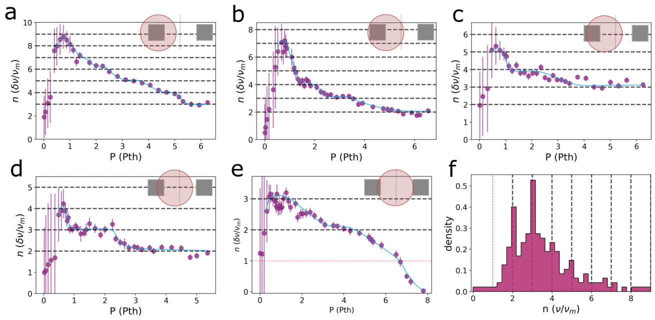

Supplementary Figure 15. **Power dependence of the inter-trap detuning for the array of 1.6μm$\times$1.6μm square traps separated by 3.2μm (a)-(e)** show the detuning between the fundamental modes of two neighbor traps, as a function of the excitation power, for different positions of the laser spot (indicated in the schemes at the top-right of each panel). Horizontal dashed lines indicate integer multiples of the fundamental phonon frequency ν_m. The error bars represent the peak's linewidth. Continuous curves are guides to the eye. **(f)** is an histogram of the frequency of occurrence of the different energy detunings, for the collection of data corresponding to the different studied laser spot positions.

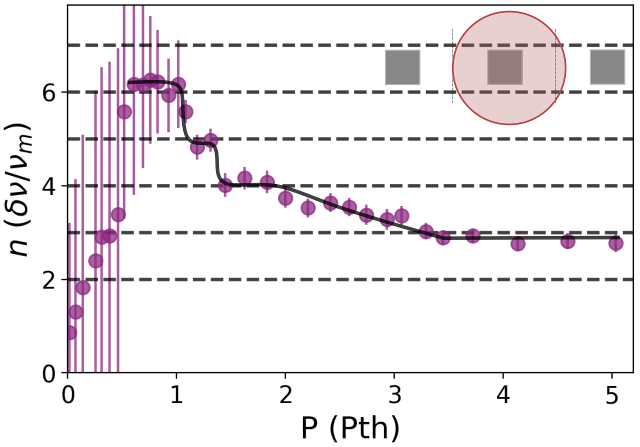

Supplementary Figure 16. **Power dependence of the inter-trap detuning for the array of 1.3μm$\times$1.3μm square traps separated by 2.6μm: almost centered excitation.** The laser spot in this experiment is almost symmetrically centered in one trap, only slightly shifted to the right. Error bars correspond to the peak's linewidth. Horizontal dashed lines indicate integer multiples of the fundamental phonon frequency ν_m. The continuous curve is a guide to the eye.

of the laser spot. moving from one trap of the array towards one of its neighbors (these are the type of experiments leading to the color maps in Fig.4 of the main text). In Figs. 15a-e the scale of the detuning is given in terms of the fundamental mechanical frequency ν_m, and integer multiples of it are indicated by the horizontal dashed lines. Several observations can be extracted from these data, namely: (i) as expected, the initial jump of the inter-trap detuning is largest when the laser spot is centered in one of the traps, and continuously decreases when a more symmetric excitation condition is set; (ii) For all cases the detuning remains finite up to the highest attained powers, except for the most symmetric case in which the spot is positioned almost at the midpoint between two traps. For this latter case, at the highest powers the two traps seem to synchronize; (iii) Suggestively, in all cases at the highest attained excitation powers, the detuning stabilizes at an integer multiple n of the fundamental mechanical frequency, $\delta\nu = \nu - \nu_0 = n \times \nu_m$. Moreover, and in coincidence with the data in Fig. 13, partial lockings signaled by a step-like behavior occur when the detuning tunes with integer multiples of the fundamental confined phonon frequency. While this intermediate frequency lockings are subtle in some cases, their existence can be further established by presenting the full set of experimental data (for the complete set of experimental runs with different spot positions) as a histogram corresponding to the frequency of occurrence of the different energy detunings. In such a histogram, shown for this set of data in Fig. 15f, steps corresponding to asynchronous locking appear as peaks. A comb of peaks appearing at 2, 3, 4 and up to 5 times ν_m is indeed quite clear in this figure.

E. Experiments on an array of 1.3μm$\times$1.3μm square traps separated by 2.6μm

In this section we present a set of experiments, similar to those presented in the previous section, but performed on an array of smaller and more closely connected square traps of 1.3μm lateral size and 2.6μm inter-trap separation. In this case the trap separation is such that the laser spot typically pumps simultaneously more than two traps. The power dependence of the inter-trap detuning, in the case of almost symmetric pumping (the spot positioned almost on top of one of the traps, but purposely slightly shifted towards the right neighbor), is shown in Fig. 16. The shown detuning corresponds to the difference in energy between the fundamental modes of the pumped trap and the neighbor at the right. Identification and filtering of the emission originated from each trap and level is performed using the spectrally resolved spatial maps as presented for these experiments in Fig. 17. Interestingly, similar to the previously shown cases, steps in the detuning dependence signaling the emergence of partial asynchronous locking can be identified at 6, 5, 4, and 3 times the phonon frequency.

As shown in Fig. 17, the emission of the centrally pumped trap is accompanied in most cases by photoluminescence from both its right and left neighbors. A detail of this feature is presented, for three representative cases, in Figs. 18(a-c). Immediately after condensation on the right trap ($P \sim 1.3P_{\rm th}$), the emission from the left trap is not observable. At $P \sim 2.0P_{\rm th}$ the left trap starts to become populated (Fig. 18a), and due to its smaller occupation its emission is clearly red-shifted compared to that from the right trap. This red-shift diminishes with increasing power (Fig. 18b), and above $P \sim 3.0P_{\rm th}$ is zero within the experimental uncertainty (Fig. 18c).

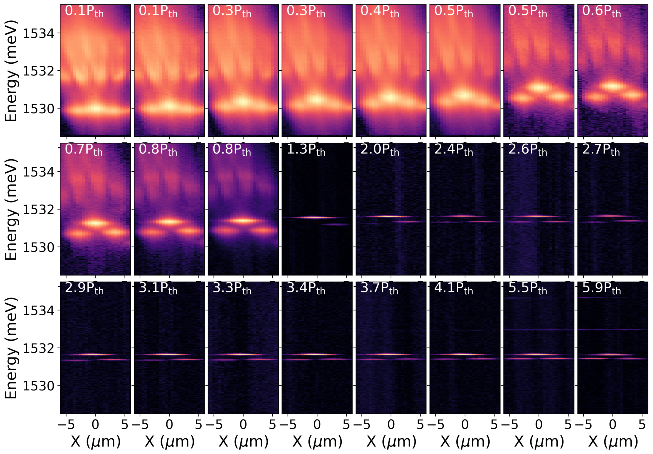

Supplementary Figure 17. **Spectrally resolved spatial images at different excitation powers for the array of 1.3μm$\times$1.3μm square traps separated by 2.6μm.** The detuning data in Supplementary Fig. 16 is obtained from the difference in energy of the fundamental s-like modes, between the pumped central trap and the neighbor one at its right.

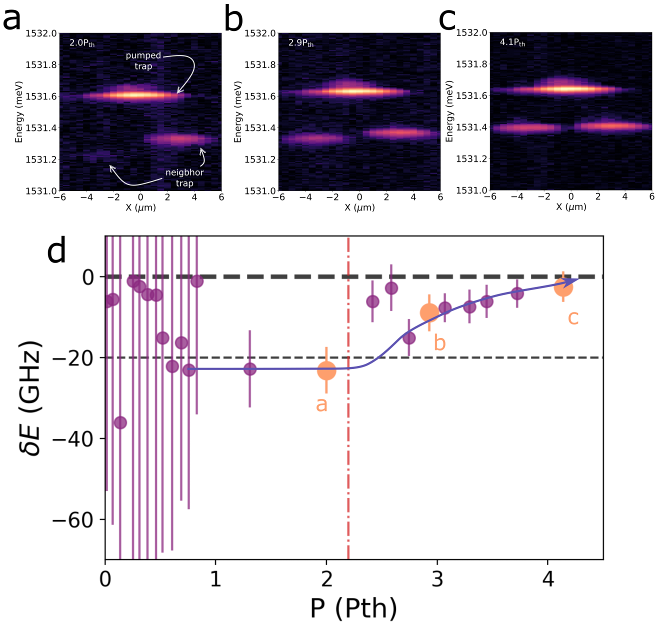

Supplementary Figure 18. **Detail of spatial images at different excitation powers for the array of 1.3μm$\times$1.3μm square traps separated by 2.6μm.** Same conditions as in Supplementary Fig. 17. **(a)-(c)** correspond to three different excitation powers. Note that due to the slight shift of the laser spot from the central trap, the right neighbor trap condenses first (panel **a**), and is blue shifted respect to the left trap. With increasing power the left trap condenses also (panel **b**), and finally both left and right traps *synchronize* at an identical detuning of $-3\nu_m$ respect to the central trap (panel **c**). The power dependence of the detuning between left and right neighbor traps is shown in **d**. The error bars in this figure represent the peak's linewidth.

That is, the left and right neighbor traps have synchronized (see Fig. 18d), and *both* lock at 3 times the phonon frequency from the central pumped trap.

Another run of experiments performed in this same array but for the laser spot position substantially shifted towards the right neighbor, is presented in Figs. 19(a-c). In this case, emission from up to three neighbor traps can be identified (see Fig. 19c). The left panel in Fig. 19a represents the detuning between these modes (labeled as 1-3) and the centrally pumped trap (identified as 0). Again, signatures of asynchronous locking at integer multiples of the phonon frequency are perceived. To strengthen this conclusion, the right panel in this figure shows the relative difference in frequency between the neighbor traps. Quite notably, all levels shift with increasing power reducing their detuning respect to the centrally pumped trap, but keeping a relative frequency separation between them very close to 3 times the phonon frequency. We note that $3\nu_m \sim 60$GHz correspond to the frequency of the first overtone of the fundamental confined vibrational mode. A calculation of the direct inter-trap coupling J for this array of more closely packed traps using the Gross-Pitaevsky modeling as in Supplementary Note 6, shows that it is not negligible. Thus, this observation might be indicative that, for this array, the direct linear optomechanical coupling between traps is playing a larger role.

F. Experiments on an array of 1.0μm$\times$1.0μm square traps separated by 2.0μm

In this section we present experiments performed on an array of the smallest and more closely packed studied traps, of 1.0μm lateral size and 2.0μm inter-trap separation. Based on the Gross-Pitaevski modelling of the trap states, it follows that this situation corresponds to that of a strongly connected array, with inter-trap interaction J of the same order of magnitude as the phonon frequency, and characterized by lattice modes that extend through the array. The experiments are presented in Figs. 20(a-d). A scheme of the positioning of the laser spot is shown in panel (a). Panel (b) presents the measured frequency detuning between all the observed modes (labeled 1-5) and the most intense higher energy one (identified as 0). The corresponding spectrally resolved spatial images are shown in panel (c), and the labeling of the modes is given in panel (d). As expected from the calculation, it is clear that here the modes extend spatially having spectral density at different traps of the array. Moreover, several of them are occupied when the condensate is formed and, most noteworthy, their relative frequency difference stabilizes with increasing excitation power at detunings that again correspond to integer multiples of the phonon frequency. It follows from the histogram in Fig. 20(b) that, indeed, modes tend to concentrate at detunings that match with multiples of the phonon frequency. Note that, in this case, the relative separation between 4 of the modes (modes 2-5) is very close to ν_m (i.e., to $n = 1$).

A final remark regarding the crystalline direction in which experiments have been performed: All but one of the experiments analyzed in the main text and this Supplementary

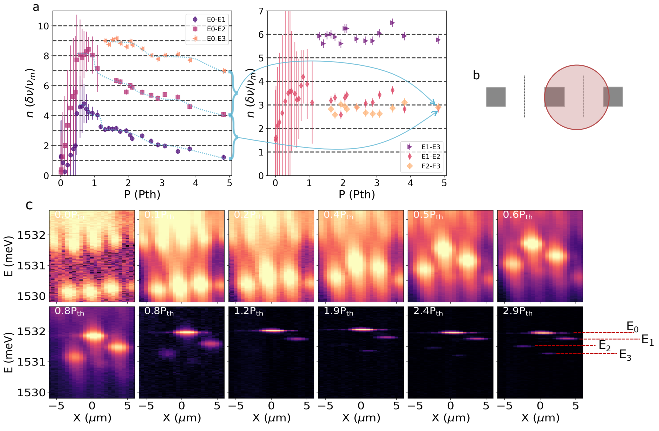

Supplementary Figure 19. **Power dependence of the inter-trap detuning for the array of 1.3μm$\times$1.3μm square traps separated by 2.6μm: non-centered excitation.** The left panel in **(a)** presents the detuning between the different observed fundamental levels (labeled as 1-3) and that of the pumped central trap (labeled as 0). The identification of the levels is given in the bottom-right-most panel of **(c)**. The right panel in **(a)** presents the relative detuning between levels 1, 2, and 3. The spot position is schematized in **(b)**. Panel **(c)** are the spectrally resolved spatial images at the different applied optical powers.

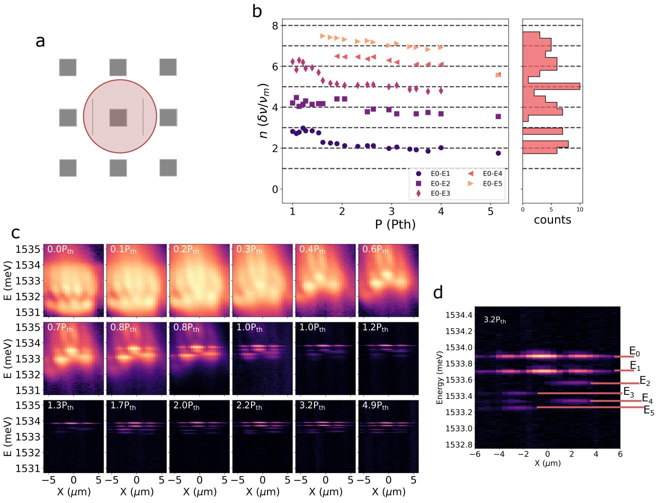

Supplementary Figure 20. **Power dependence of the inter-trap detuning for the array of** 1.0μ**m**$\times 1.0\mu$**m square traps separated by** 2.0μ**m.** The spot position is shown in **(a)**. Panel**(b)** presents the detuning between the different observed fundamental levels (labeled as 1-5) and that of the highest energy one (labeled as 0). Panel **(c)** are the spectrally resolved spatial images at the different applied optical powers. The identification of the levels is given in **(d)**.

Notes were performed collecting the emitted light with the traps aligned along the X crystalline direction, i.e. the [1 -1 0], as defined in Fig.1(a) of the main text. The only experiment shown with the traps aligned along the Y direction ([1 1 0]) is shown in Fig. 5 of the main text. We do not observe fundamental differences when collecting in either of the two directions.

G. Method for the construction of the histogram in Fig. 4 of the main text

The histogram shown in Fig. 4 of the main text, summarizes the complete set of experiments presented in the last three sections, performed on arrays of traps of 1.6μm, 1.3μm, and 1.0μm lateral size. These arrays span situations in which, as explained above, the lattice separation is such that only the quadratic inter-trap optomechanical coupling is significant (1.6μm), or both linear and quadratic contributions are present (1.0μm). The presented histogram of frequency of occurrence of inter-trap detunings provides, to the best of our understanding, the most compelling evidence of the existence of the proposed asynchronous locking. We will thus explain here the procedure and assumptions involved in the obtention of this figure.

The procedure used can be followed in Figs. 21(a-c). If all measured detunings are pluged in the histogram of the frequency of their occurrence without any further processing, we obtain the histogram shown in Fig. 21(a). We recall that in this histogram we are including only the detunings between the observed secondary modes and the most intense higher energy one, and *not* the detunings corresponding to the relative frequency difference between secondary modes. This crude version of the histogram already shows clear peaks at $n = 2, 3$ and 4, and possibly though less clear at $n = 5$, the multiples higher than this being washed out by broadening. We have noted from the long series of experiments performed that, when partial and final lockings are attained, the phonon frequency that better explain the complete set of steps observed is not exactly the same for the different arrays, nor even when centrally pumping different traps of the same array. All the optimum values are around a value of $\nu_m = 20.6$ GHz, but with a small dispersion of around $\sigma \sim 0.8$ GHz. This difference is not critical for the lower multiples n (and consequently the peaks are clear in Fig. 21(a)), but contributes to a significant broadening for the larger multiples $n\nu_m$, obscuring the presence of the corresponding peaks in the histogram. A possible origin for such small variation of confined mode energy can have several explanations. The frequency of the modes can depend on the precise geometry of the traps, and also on the trap separation. Also they can depend on the presence of residual strain, which can vary for the different arrays and for different traps within an array. It can not be excluded that a small change of mode frequency is induced by the optical pumping (so-called optical-spring effect). Last but not least, the observed $\sigma \sim 0.8$ GHz is in the limit of our spectral resolution.

In view of the above considerations, if we allow for a small adjustment of the involved mechanical frequencies, and do not directly count the occurrence of certain detunings measured in GHz but in multiples of the corresponding phonon frequency (which is taken the same within an experiment for all excitation powers, but not necessarily identical for different arrays or spot positions in an array), we obtain the histogram presented in Fig. 21(b). Quite notably, and as expected, this tiny adjustment leads to a better definition of the peaks, which now become narrower and clearly discernible up to $n = 7$.

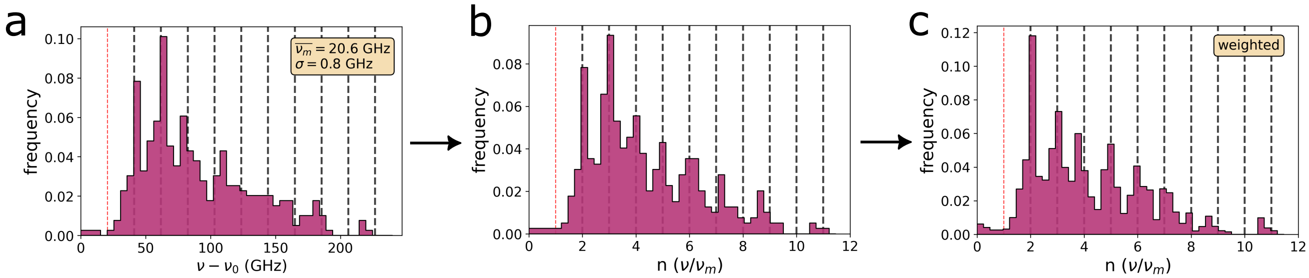

Supplementary Figure 21. **Procedure to obtain the histograms of occurrence of different inter-level detunings.** The histogram in **(a)** is obtained counting the frequency of occurrence of certain detunings (as discussed in Figs. 15, 16, 19 and 20), including in the data set all the experiments performed for the different spot positions and for the three studied arrays (traps of 1μm, 1.3μm, and 1.6μm lateral size). The vertical lines are equispaced by 20.6 GHz. This latter value is the average of the phonon energy as determined from the step separation in the different experiments. The dispersion of the values is $\sigma \sim 0.8$ GHz. If the value of phonon energy that best fits the step separation is used for each experiment, the histogram in **(b)** is obtained. Note that in this case the detuning is not given in GHz but in the number of involved phonons n. The histogram in **(c)** accounts for the different density of experimental points taken in different applied power regions. This is done by weighting the counting of each point by the average power difference between the point under consideration, and the ones taken with applied powers just below and just above.

A final processing of the data follows from noting that, in the experiments, the excitation power is finely tuned but not always keeping a constant step. This is particularly true for the higher optical powers, because once the peak energies start to saturate the data taken become more sparse. This contributes to a distortion of the relative amplitude of the peaks in the histograms as presented in Figs. 21(a) and (b). To better represent a precise frequency of occurrence of a certain detuning, and similar to a figure representing a density of states, the data should be weighted to represent equally spaced excitation power steps. We do this by multiplying each occurrence by an average of the laser power difference between the counted experiment and the consecutive ones with powers just below and above it. When this is done we obtain Fig. 21(c), which is the version presented in Fig. 4 of the main text. As compared to Fig. 21(b) the information concerning the presence or absence of peaks in the histogram is the same, but the relative amplitude of the occurrence of the different multiples has become physically more meaningful.

SUPPLEMENTARY NOTE 8: MODEL FOR THE LOCKING OF THE MODES

In this section we briefly discuss some possible asynchronous locking mechanics with a minimalist modelling. The main point we would like to emphasize is that asynchronous locking is an ubiquitous property of coupled non-linear harmonic oscillators when some of the parameters are modulated in a time periodic manner. In our case, all cavity parameters are intertwined with well defined phonons modes, hence providing a natural source for such modulations when excited. In the following, building upon the well-known case of polariton synchronization in the static case we present a step by step scenario to account for the observed phenomena. Namely, we will consider first the case where the time dependent modulation is given and later discuss how a self-consistent treatment of the polariton and phonon modes using the model described in Eq. (17) can lead to it.

A. Static synchronization

Here we revisit the static synchronization as discussed by Wouters [30]. Assuming a simple scenario, we describe two coupled polariton modes ($j = 1, 2$) and the corresponding reservoir's densities as

$$\begin{aligned} i\hbar\dot{\psi}_j &= (\varepsilon_j + U_j|\psi_j|^2 + U_j^{\rm R} n_j)\psi_j - J\psi_{3-j} + \\ &+\frac{i\hbar}{2}(R n_j - \gamma)\psi_j \, , \\ \dot{n}_j &= P_j - \gamma_{\rm R}\, n_j - R|\psi_j|^2 n_j \, . \end{aligned} \tag{18}$$

Here ε_j is the bare energy of the j-mode, U_j and $U_j^{\rm R}$ are the polariton-polariton and polariton-reservoir interaction couplings, J describes a direct hopping term between modes, γ the polariton decay and R the stimulated loading from the reservoir. The dynamics of the later is controlled by the pump power P_j, the excitonic decay rate $\gamma_{\rm R}$ and the stimulated decay to the condensate. We notice here that since we are considering two separated modes (on different traps) the direct overlap between them is assumed small and so the equation for the local value of the reservoir density only depends on the amplitude of the corresponding polariton mode. A more involved model, including a cross term (as would be the case for a single trap or strongly coupled traps) could be easily included. In that case, synchronisation due to the competition of the reservoir-mediated population of the modes is also possible [31].

We seek for a synchronized solution of the form $\psi_j = \sqrt{\rho_j} e^{-i\omega t} e^{\pm i\theta/2}$ (here the $+$ sign corresponds to $j = 1$) and $\dot{n}_j = 0$. Introducing this ansatz in Eqs. (18) we get the

following set of algebraic equations

$$\begin{aligned}
\frac{1}{\alpha}\frac{(\xi_1^0-\xi_1)}{\xi_1+1} &= -2J_g\sin(\theta)\,, \\
\alpha\frac{(\xi_2^0-\xi_2)}{\xi_2+1} &= 2J_g\sin(\theta)\,, \\
\alpha J\cos(\theta)+\hbar\omega &= \varepsilon_1+\frac{U_1^{\rm R}n_0\left(\xi_1^0+1\right)}{\xi_1+1}+\xi_1\rho_0U_1\,, \\
\frac{J\cos(\theta)}{\alpha}+\hbar\omega &= \varepsilon_2+\frac{U_2^{\rm R}n_0\left(\xi_2^0+1\right)}{\xi_2+1}+\xi_2\rho_0U_2\,,
\end{aligned} \tag{19}$$

where $\alpha = \sqrt{\rho_2/\rho_1}$, and we have introduced some dimensionless parameters so that: $J_g = J/\hbar\gamma$, $\xi_j = \rho_j/\rho_0$, $\rho_0 = \gamma_{\rm R}/R$, $P_j = (1+\xi_j^0)P_{\rm th}$, $P_{\rm th} = \gamma\gamma_{\rm R}/R$, $n_0 = \gamma/R$. We also made use of the stationary solution for the reservoir, $n_j = P_j/(\gamma_{\rm R}+R\rho_j) = n_0\left(\xi_j^0+1\right)/(\xi_j+1)$.

Equations (19) need to be solved for ξ_j, ω and θ. Notice that the first two allow for the determination of $\xi_j(\theta)$, independently of the interactions (for instance $J=0$ implies $\xi_j = \xi_j^0$). While it is possible to find a general solution for $\xi_j(\theta)$, it involves a quartic polynomial with generic roots and, in practice, it is in general a bit cumbersome to determine the one with physical meaning ($\xi_j, \alpha > 0$).

However, an analytical condition for θ can be found if one assumes that $R\rho_j \gg \gamma_{\rm R} \to \xi_j \gg 1$, i.e. $P_j \gg P_{\rm th}$, deep into the condensed regime [30]. In that case, $\xi_j + 1 \to \xi_j$ in the above equations (and for consistency the same applies to ξ_j^0). Hence, from the first two equations in Eq. (19) one gets

$$\begin{aligned}
\xi_1(\theta) &= \Theta(-\theta)g(\theta)+\Theta(\theta)\frac{(\xi_1^0)^2}{(1+4J_g^2\sin^2(\theta))g(\theta)}\,, \\
\xi_2(\theta) &= \frac{\left(\xi_1(\theta)-\xi_1^0\right)\xi_2^0}{4J_g^2\xi_1(\theta)\sin^2(\theta)+\xi_1(\theta)-\xi_1^0}\,,
\end{aligned} \tag{20}$$

with

$$\begin{aligned}
g(\theta) &= (\xi_1^0)^2\Big\{2J_g\sin^2(\theta)\left(\sqrt{\xi_1^0\xi_2^0\csc^2(\theta)+J_g^2\left(\xi_1^0+\xi_2^0\right)^2}\right. \\
&\left.+J_g\left(\xi_1^0+\xi_2^0\right)\right)+\xi_1^0\Big\}^{-1}\,,
\end{aligned} \tag{21}$$

and $\Theta(x)$ the step function. The final equation that determines θ is then

$$\begin{aligned}
\Delta\varepsilon &= f(\theta)\,, \\
&= J\cos(\theta)\left(\frac{1}{\alpha(\theta)}-\alpha(\theta)\right) \\
&+\frac{U_1^{\rm R}n_0\xi_1^0}{\xi_1(\theta)}+U_1\rho_0\xi_1(\theta)-\frac{U_2^{\rm R}n_0\xi_2^0}{\xi_2(\theta)}-U_2\rho_0\xi_2(\theta)\,,
\end{aligned} \tag{22}$$

with $\Delta\varepsilon = \varepsilon_2 - \varepsilon_1$. Once θ is determined, the locking energy is given by

$$\hbar\omega = \frac{\bar\varepsilon_1+\bar\varepsilon_2}{2}-\frac{J\cos(\theta)}{2}\left(\alpha+\frac{1}{\alpha}\right)\,, \tag{23}$$

and since

$$\bar\varepsilon_2-\bar\varepsilon_1 = J\cos(\theta)\left(\frac{1}{\alpha}-\alpha\right)\,, \tag{24}$$

we get

$$\hbar\omega = \bar\varepsilon_1 - \alpha\,J\cos(\theta)\,. \tag{25}$$

Here $\bar\varepsilon_j = \varepsilon_j + U_j\rho_0\xi_j + U_j^{\rm R}n_0\frac{\xi_j^0}{\xi_j}$ is the dressed energy. Notice that we allowed here for different pump powers on each trap, $\xi_1^0 \neq \xi_2^0$. The resulting phase diagram for the synchronization is identical to the one reported in Ref. [30] when $\xi_1^0 = \xi_2^0$. When $\xi_1^0 \neq \xi_2^0$ the diagram remains very similar except that $\Delta\varepsilon$ is not longer symmetric around zero.

B. Mechanical wave induced energy locking

Equations (18) can be generalized to account for the presence of mechanical waves by assuming that some of the parameter, say ε_i or J, are modulated in time. Modulation of the site energies could arise from s-like cavity phonons while the modulation of the coupling J can be due to p-like phonons through excited states (cf. Eq. (17) and discussion below).

To illustrate the simplest situation where asynchronous locking appears, let us consider the case when the coupling constant is time dependent, $J(t)$, being modulated by a coherent mechanical wave of frequency Ω. While in general solutions for the dynamics can only be obtained numerically, we can already grasp the presence of a locking phase by making use of the rotating wave approximation (RWA). In this case, assuming $\varepsilon_1 \sim \varepsilon_2 + \hbar\Omega$, we have

$$\begin{aligned}
i\hbar\dot\psi_1 &\simeq (\varepsilon_1+U_1|\psi_1|^2+U_1^{\rm R}\,n_1)\psi_1-\left(Je^{i\Omega t}\right)^*\psi_2+ \\
&+\frac{i\hbar}{2}(R\,n_1-\gamma)\psi_1\,, \\
i\hbar\dot\psi_2 &\simeq (\varepsilon_2+U_2|\psi_2|^2+U_2^{\rm R}\,n_2)\psi_2-Je^{i\Omega t}\psi_1+ \\
&+\frac{i\hbar}{2}(R\,n_2-\gamma)\psi_2\,.
\end{aligned} \tag{26}$$

It is clear from the above equations that we can follow the same procedure of the previous section, except that we now propose $\psi_1 = \sqrt{\rho_1}e^{-i\omega t}e^{i\theta/2}$ and $\psi_2 = \sqrt{\rho_2}e^{-i(\omega-\Omega)t}e^{-i\theta/2}$ as the solution for the asynchronous mechanically-induced locking phase. The resulting equations are the same as before except that, in all equations, ε_2 needs to be replaced by $\varepsilon_2 + \hbar\Omega$. Hence, the condition for obtaining a solution is given by

$$\bar\varepsilon_2-\bar\varepsilon_1 = -\hbar\Omega + J\cos(\theta)\left(\frac{1}{\alpha}-\alpha\right)\,, \tag{27}$$

with, as before, $\bar\varepsilon_j = \varepsilon_j + U_j\rho_0\xi_j + U_j^{\rm R}n_0\frac{\xi_j^0}{\xi_j}$. Note that Eqs. (20) and (21) remain valid. We therefore expect that, in the presence of a mechanical modulation of the hopping parameter, the locking of the frequency of the polariton modes at Ω to be as robust as the usual synchronization in the static case.

To further proceed beyond the RWA, we made full numerical simulations from where the stationary solutions of Eqs.(26) were obtained. We first consider the presence

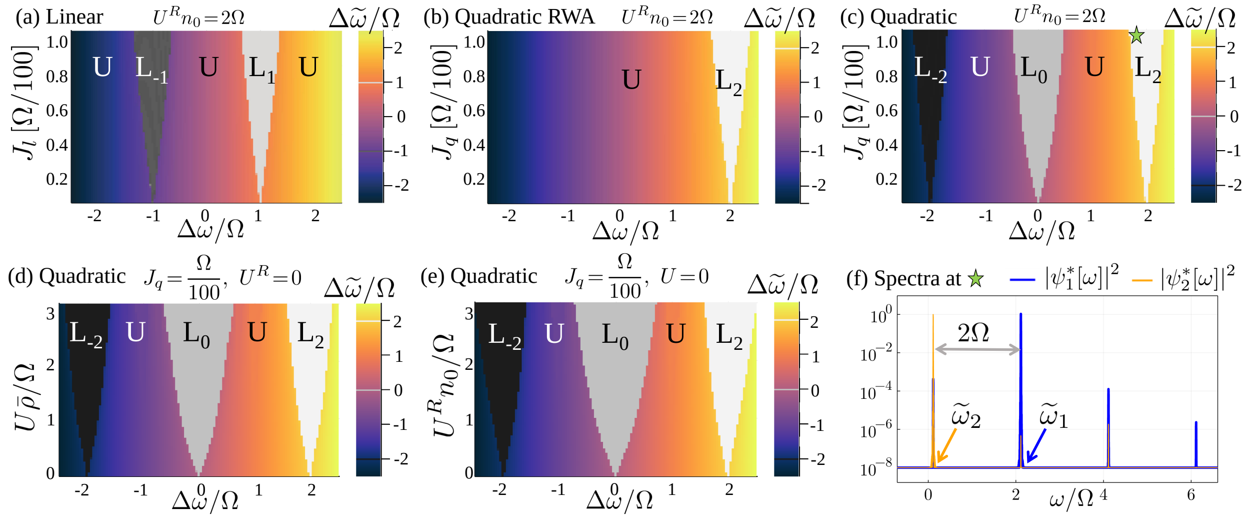

Supplementary Figure 22. **Interaction-enhanced intermode frequency locking of the steady state subject to a phonon-induced** $J(t)$**.** Color-maps of the computed dressed detuning $\Delta\widetilde{\omega}$, as a function of the bared detuning, $\Delta\omega$ and some of the physical parameters of the model for $\kappa = 0.2\Omega$, $\bar{\rho} = 10^5$ and $J(t)$ corresponding to the linear case $J_l(e^{i\Omega t} + e^{-i\Omega t})$ (a), the quadractic-RWA $J_q e^{-i2\Omega t}$ (b), or full quadratic $J_q(e^{i2\Omega t} + e^{-i2\Omega t} + 2)$ (c). In panels (a-c) we take $U = 0$ and $U^{\mathrm{R}} n_0 = 2\Omega$ and change the phonon coupling strengths, J_l or J_q. The dressed frequencies of the two modes get *locked* in the regions labeled as L_n having constant $\Delta\widetilde{\omega} = n\Omega$: These are regions centered at values of the bare detuning $\Delta\omega = n\Omega$. At the remaining regions, labelled as U, the obtained $\Delta\widetilde{\omega}$ is *unlocked*. Panels (d) and (e) show, for the full quadratic case with $J_q = 0.01\Omega$, that both U and U^{R} enhance the possibility of locking even for J_q of the order of one percent of the phonon frequency Ω. (f) polariton power spectra of the steady state (normalized with $\bar{\rho}$) for the locked condition shown in panel (c). The main spectral peaks at $\widetilde{\omega}_j$ present 2Ω-separated sidebands having spectral weight that are around four orders of magnitude, or more, smaller than the main peak.

of a single phonon mode of frequency Ω and analyse three different forms for $J(t)$: i) $J(t) = J_q\, e^{i\Omega t}$, corresponding to a generic case in the RWA; ii) an optomechanical quadratic coupling in the phonon displacement, $J(t) = J_q\,(e^{i2\Omega t} + e^{-i2\Omega t} + 2)$ with $J_q = g^{(2)} n_b$; and iii) a linear coupling $J(t) = J_l\,(e^{i\Omega t} + e^{-i\Omega t})$ with $J_l = g^{(1)}\sqrt{n_b}$. The linear and quadratic terms follow from assuming the presence of a coherent population of $n_b \gg 1$ phonons, i.e., $b(t) + b(t)^* = 2\sqrt{n_b}\cos(\Omega t)$, and an OM interaction having a hopping term between the two polariton modes with a prefactor containing the m-order power of the phonon displacement, namely, $-\hbar g^{(m)}(\hat{b} + \hat{b}^\dagger)^m$ (cf. Eq. (17) for the quadratic case).

As in the last section, we work well above the condensation threshold where $R|\psi_j|^2 \gg \gamma_{\mathrm{R}}$. Then $n_j = n_0\bar{\rho}/|\psi_j|^2$ with equal power for both modes (the unbalance of the pump power on each trap is absorbed in the asymmetric blue shift described by the bare energy difference $\varepsilon_1 - \varepsilon_2$). Here, for the sake of simplicity, we introduced $\bar{\rho} = P/\gamma = \rho_0\,(P/P_{\mathrm{th}}) \sim \rho_0\xi^0$. As both modes are driven to $\bar{\rho}$ we replace $(Rn_j - \gamma)$ by $\gamma(\bar{\rho}/|\psi_j|^2 - 1)$ in Eqs.(26). We choose identical interaction strengths: $U_1 = U_2 = U$ and $U_1^{\mathrm{R}} = U_2^{\mathrm{R}} = U^{\mathrm{R}}$. Therefore the reservoir-polariton and the polariton-polariton interaction induce a blue shift for mode j of $U^{\mathrm{R}} n_j = U^{\mathrm{R}} n_0(\bar{\rho}/|\psi_j|^2)$ and $U|\psi_j|^2 = U\bar{\rho}\,(|\psi_j|^2/\bar{\rho})$, respectively. In the results below we vary the quantities $U^R n_0$ and $U\bar{\rho}$, corresponding to the expected blue shifts for $|\psi_j|^2 = \bar{\rho}$.

Our goal is to study the tendency of the two modes to get locked, i.e., presenting frequencies separated by fixed amounts related to the phonon frequency. This requires the appearance of large areas in terms of the bare detuning, $\Delta\omega \equiv (\varepsilon_1 - \varepsilon_2)/\hbar$, in which the dressed detuning is locked. In order to find the dressed detuning in a operative way from the simulated steady state we define $\Delta\widetilde{\omega} \equiv \widetilde{\omega}_1 - \widetilde{\omega}_2$, with the dressed frequencies $\widetilde{\omega}_j$ given by the frequencies having the greatest spectral weight in $\psi_j^*[\omega]$, i.e., the Fourier transform of each mode amplitude, $\psi_j^*(t)$.

In Fig. 22 we present and describe the results of our simulations showing how the interactions enhance the area of the locked regions. In panels (a-c) we set $U = 0$ and simulate the effect of $U^{\mathrm{R}} n_0$ being of the order of $\hbar\Omega$. This choice is due to the dominant photon-like character of the measured polaritons making the reservoir-polariton coupling $U^{\mathrm{R}} n_0$ larger than the polariton-polariton coupling $U\bar{\rho}$; this is consistent with our Gross-Pitaeskii equation simulations [27]. However, we note that as is expected from the analytical theory of the previous section, similar enhancement of the locked regions is observed for $U^{\mathrm{R}} = 0$ and $U \neq 0$ (see Fig. 22(d)). Importantly, panels (a-e) show that even for J_q or J_l of one percent of $\hbar\Omega$ the locked regions acquire a significant portion of the parameter space. We can also see, from comparing Fig. 22(b) with Fig. 22(c), that at the simulated moderate values of the parameters J_q, $U\bar{\rho}$, and $U^{\mathrm{R}} n_0$ the RWA is enough to describe the limits of the L_2 locked area fairly well, i.e., the region having dressed detuning $\Delta\widetilde{\omega} = 2\Omega$. For larger values of the relevant parameters the RWA breaks down as other locked situations overlap. We note that the linear coupling case, Fig. 22(a), presents its dominant locked regions at $\Delta\widetilde{\omega} = \pm\Omega$. Finally, it is worth pointing out that inside the locked region the power spectrum of the modes (Fig. 22(f)) shows sidebands that are several orders of magnitude smaller than the main peak and

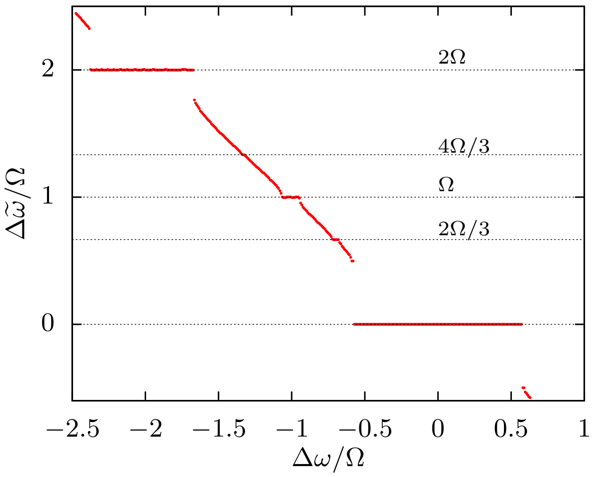

Supplementary Figure 23. **Non-linear effects on locking.** In the presence of non-linear terms, as the ones introduced by the interactions, asynchronous locking can occur at fractional values of the mechanical wave frequency Ω. The plot here shows the dressed detuning $\Delta\widetilde{\omega}$ as a function of the bare detuning $\Delta\omega$ for the quadratic case presented in Fig. 22(c) with $J_q = 0.01\hbar\Omega$. Note that, apart from the expected lockings at 2Ω and 0, it is apparent that there is a significant locking region at Ω, as well as smaller ones at $4\Omega/3$, and $2\Omega/3$.

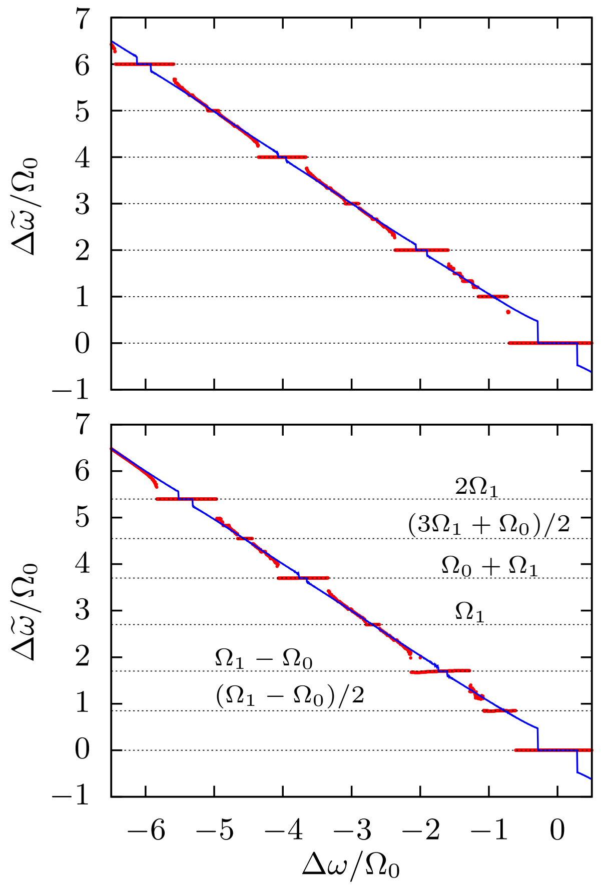

Supplementary Figure 24. **Locking in the presence of two phonons.** Dressed detuning $\Delta\widetilde{\omega}$ as a function of the bare detuning $\Delta\omega$ for the case of three polaritons modes coupled to two mechanical waves of frequency Ω_0 and Ω_1 [cf. Eqs. (15) and (16) with $b_m + b_m^\dagger \to A_m \cos(\Omega_m t)$]. Panel (a) shows the case $\Omega_1 = 3\Omega_0$ (experimental situation) and panel (b) the one $\Omega_1 = 2.7\Omega_0$. Red dots correspond to $U^R n_0 = 0.6\hbar\Omega_0$ and the solid blue line to the case $U^R = 0$. Note that the non-interacting case shows locking only at $\Delta\widetilde{\omega}/\Omega_0 = 0, \Omega_1 \pm \Omega_0$ and $2\Omega_1$ and that in the experimental relevant situation (a) there are frequency lockings at every $n\Omega_0$. Here we used : $A_n = \Omega_n/g_n$, $\Delta = 25\Omega_0$, $P_i = 3P_{\rm th}$,$\gamma = 2\times10^{-2}\Omega_0$, $\Gamma = 5\times10^{-4}\Omega_0$.

hence extremely hard to resolve experimentally.

Although not apparent in the color maps of Fig. 22, a closer detailed look revels the presence of asynchronous locking regions at fractional values of the driving frequency, that is, subharmonic locking. This is clearly seen in Fig. 23, which corresponds to $J_q = 0.01\hbar\Omega$ in Fig. 22(c). The fractional values arise from the non-linear terms being, in particular, strongly controlled by the interactions—they are hardly detected for such small value of J_q when both U^R and U are zero.

The phase diagram of possible locking frequencies becomes even richer when a second phonon of a different frequency is included, as for instance the 60 GHz p-like phonon. The corresponding model has been already introduced in Eqs. (15) and (16) or equivalently, through its low energy approximation Eq. (17). The equations of motion obtained from there for the polariton fields [27] are solved here for a given phonon amplitude by taking $x_m = b_m + b_m^\dagger \to A_m \cos(\Omega_m t)$. This corresponds to modulate $J(t)$ in Eq. (26) with the sum of two quadratic terms with frequencies Ω_1 and Ω_2 and a third term with the product of two linear modulations of the same frequencies—in addition to a small modulation of the site energies.

The numerically computed dressed detuning $\Delta\widetilde{\omega}$ is shown in Fig. 24 with and without interactions in the case of $\Omega_1 = 3\Omega_0$ (a) and $\Omega_1 = 2.7\Omega_0$ (arbitrarily chosen not to be an integer multiple of Ω_1) (b). Locking regions clearly develop at $2\Omega_i$, $\Omega_1 \pm \Omega_0$ and $(\Omega_1 - \Omega_0)/2$, to mention a few prominent cases. When the two phonon frequencies become commensurate, as in the experimentally relevant case of the 20 and 60 GHz phonons, more than one process (absorption or emission of a phonon) can contribute/compete to the establishing of the locking (for instance, the $2\Omega_0$ and the $\Omega_1 - \Omega_0$ processes). This particular relation between frequencies leads to the appearance of locking regions near every integer multiple of Ω_0 (as observed in the experiments, cf. Fig. 4 on the main text). When Ω_1 is not commensurate with Ω_0 a more complex pattern emerges being determined by the relative intensity of the two mechanical waves.

C. Self-consistent mechanical wave

So far we have assumed that the mechanical wave (phonons) where present in the system. However, an important question remains, how are they generated in the first place? In Ref. [27] we argued that the equations of motion derived from, say, Eq. (17), including the ones for

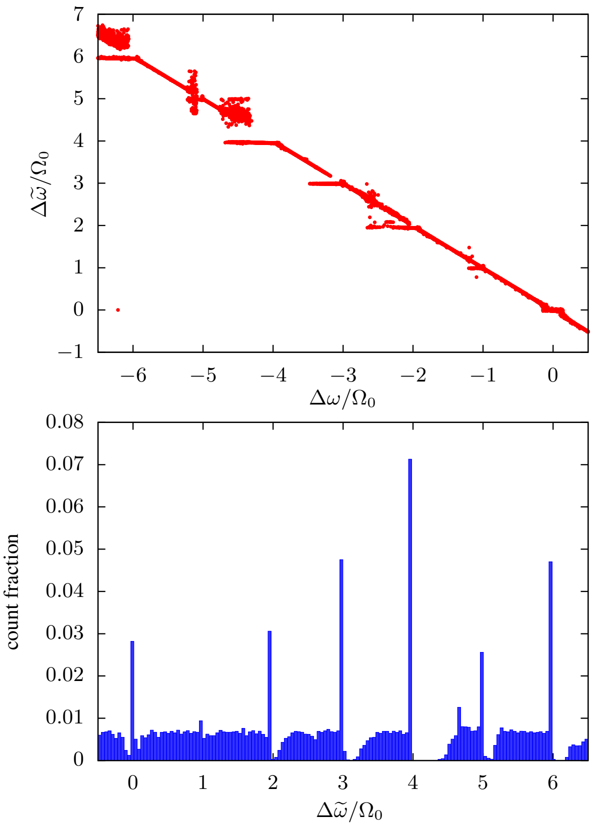

Supplementary Figure 25. **Self-consistent locking.** Panel (a) Same as the previous figure but where the dynamics of the phonons is calculated self consistently using Eq. (28) with a large initial condition, $x_m \sim \Omega_m/g_m$. A noisy and unstable behavior is apparent from the figure but locking at $n\Omega_0$ are clearly visible. The appearance of such well defined locking is emphasized in the histogram presented in panel (b). Here $g_0 = g_1/3 = 10^{-3}\Omega_0$, $U^R n_0 = U\rho_0 = 0.6\hbar\Omega_0$ and $\gamma = 4\times 10^{-3}\Omega_0$.

the phonons, leads to a parametric instability that originates a large amplitude of the phonon field for certain particular detunings ($2\Omega_0$ and $4\Omega_0$ in the case $\Omega_1 = 3\Omega_0$), as experimentally observed in [10]. Indeed, the equation for the phonons reads

$$\begin{aligned}\ddot{x}_n &= -\Gamma_n \dot{x}_n - \Omega_n^2 x_n \\ &\quad -4\Omega_n \sum_{j=1}^{2}\sum_m \frac{g_{jn}g_{jm}}{\Delta}\left(a_3^* a_3 - a_j^* a_j\right) x_m \\ &\quad +4\Omega_n \sum_m \frac{g_{1n}g_{2m}}{\Delta}\left(a_1^* a_2 + a_2^* a_1\right) x_m \,, \qquad (28)\end{aligned}$$

that corresponds to a set of coupled harmonic oscillators with parametric driving (by the polariton modes). The latter becomes evident when noticing that the last two terms, for $m = n$, correspond to a time dependent modulation of the phonon frequency. Moreover, the last term can become resonant at appropriate inter-trap detunings. It is important to keep in mind that this modulation, provided by the polariton modes, is not given but rather obtained self-consistently by solving all the equations of motion simultaneously [27]. This is particularly relevant when interactions are included as to obtain fractional locking—not included in the analysis of Ref. [27].

While this approach is able to capture the presence of instability regions, the size of the latter strongly depends on the seed of the phonon field, and hence they are rather narrow when the starting amplitude of the phonon field is small (say $x_m \ll \Omega_m/g_m$). However, since our model is an oversimplification of a very complex system, different mechanism not fully considered here (highly excited states, phonon emission through relaxation mechanisms, reservoir feedback dynamics, etc) could in principle lead to some large fluctuation of the phonon field. In fact, the experimental results of Ref. [10] suggest that this is the case as they show that previous to the appearance of well-defined symmetric sidebands in the PL spectrum, a signature of a coherent phonon field, phonon-induced PL satellites peaks appear. The latter is an indication of the presence of a large number of phonons in the system. Notably, if we assume that that is the case and introduce in our equations a large initial condition (say, $x_m \sim \Omega_m/g_m$) a much wider instabilities appear. The corresponding calculated dressed detuning $\Delta\tilde{\omega}$ shows now large fluctuations with locking features at all the experimentally observed detunings, as shown in Fig. **25**, together with the corresponding histogram as described in the main text. Here, in order to better stabilize the odd lockings, the polariton-polariton interaction was included as well as some gaussian random noise added to the Gross-Pitaeskii equation.

[1] O. El Daif, A. Baas, T. Guillet, J.-P. Brantut, R. I. Kaitouni, J. L. Staehli, F. Morier-Genoud, and B. Deveaud, Polariton quantum boxes in semiconductor microcavities, Appl. Phys. Lett. **88**, 061105 (2006).

[2] K. Winkler, J. Fischer, A. Schade, M. Amthor, Robert Dall, Jonas Gessler, M. Emmerling, E. A. Ostrovskaya, M. Kamp, C. Schneider, and S. Höfling, A polariton condensate in a photonic crystal potential landscape, New J. Phys. **17**, 023001 (2015).

[3] A. S. Kuznetsov, P. L. J. Helgers, K. Biermann, and P. V. Santos, Quantum Confinement of Exciton-Polaritons in Structured (Al,Ga)As Microcavity, Phys. Rev. B **97**, 195309 (2018).

[4] P. Sesin, S. Anguiano, A. E. Bruchhausen, A. Lemaître, and A. Fainstein , Cavity Optomechanics with a Laser Engineered Optical Trap, Phys. Rev. B **103**, L081301 (2021).

[5] S. Anguiano, A. E. Bruchhausen, B. Jusserand, I. Favero, F. R. Lamberti, L. Lanco, I. Sagnes, A. Lemaître, N. D. Lanzillotti-Kimura, P. Senellart, and A. Fainstein, Micropillar Resonators for Optomechanics in the Extremely High 19-95 GHz Frequency Range, Phys. Rev. Lett. **118**, 263901 (2017).

[6] S. Anguiano, A. E. Bruchhausen, I. Favero, I. Sagnes, A. Lemaître, N. D. Lanzillotti-Kimura, and A. Fainstein, Optical cavity mode dynamics and coherent phonon generation in high-Q micropillar resonators, Phys. Rev. A **98**, 013816 (2018).

[7] C. Thomsen, H. T. Grahn, H. J. Maris, and J. Tauc, Surface generation and detection of phonons by picosecond light pulses, Phys. Rev. B **34**, 4129 (1986).

[8] O. Matsuda, T. Tachizaki, T. Fukui, J. J. Baumberg, and O. B. Wright, Acoustic phonon generation and detection in GaAs/ $Al_{0.3}Ga_{0.7}$ As quantum wells with picosecond laser pulses, Phys. Rev. B **71**, 115330 (2005).

[9] A. Fainstein, N. D. Lanzillotti-Kimura, B. Jusserand, B. Perrin, Strong optical-mechanical coupling in a vertical GaAs/AlAs microcavity for subterahertz phonons and near-infrared light, Physical Review Letters **110**, 037403 (2013).

[10] D. L. Chafatinos, A. S. Kuznetsov, S. Anguiano, A. E. Bruchhausen, A. A. Reynoso, K. Biermann, P. V. Santos, and A. Fainstein, Polariton-driven phonon laser, Nature Communications **11**, 4552 (2020).

[11] M. F. Pascual-Winter, A. Fainstein, B. Jusserand, B. Perrin, and A. Lemaître, Phonon optical generation and detection spectral responses in superlattices, Rev. B **85**, 235443 (2012).

[12] P. Ruello and V. E. Gusev, Physical mechanisms of coherent acoustic phonons generation by ultrafast laser action, Ultrasonics **56**, 21 (2015).

[13] N. D. Lanzillotti-Kimura, A. Fainstein, A. Huynh, B. Perrin, B. Jusserand, A. Miard, and A. Lemaître, Coherent generation of acoustic phonons in an optical microcavity, Phys. Rev. Lett. **99**, 217405 (2007)

[14] N. D. Lanzillotti-Kimura, A. Fainstein, B. Perrin, and B. Jusserand, Theory of Coherent Generation and Detection of THz acoustic phonons using Optical Microcavities, Phys. Rev. B **84**, 064307 (2011).

[15] F. R. Lamberti, Q. Yao, L. Lanco, D. T. Nguyen, M. Esmann, A. Fainstein, P. Sesin, S. Anguiano, V. Villafañe, A. Bruchhausen, P. Senellart, I. Favero, and N. D. Lanzillotti-Kimura, Optomechanical properties of GaAs/AlAs micropillar resonators operating in the 18 GHz range, Opt. Express **25**, 24437 (2017).

[16] S. Anguiano, P. Sesin, A. E. Bruchhausen, F. R. Lamberti, I. Favero, M. Esmann, I. Sagnes, A. Lemaître, N. D. Lanzillotti-Kimura, P. Senellart, and A. Fainstein, Scaling rules in optomechanical semiconductor micropillars, Phys. Rev. A **98**, 063810 (2018).

[17] M. Trigo, A. Bruchhausen, A. Fainstein, B. Jusserand, and V. Thierry-Mieg, Confinement of Acoustical Vibrations in a Semiconductor Planar Phonon Cavity, Phys. Rev. Lett. **89**, 227402 (2002).

[18] See www.comsol.com for details on this commercial general-purpose simulation software for modeling.

[19] S.Adachi, GaAs, AlAs, and $Al_xGa_{1-x}As$: Material parameters for use in research and device applications, J. Appl. Phys. **58**, R1 (1985).

[20] A. E. Bruchhausen, N. D. Lanzillotti-Kimura, B. Jusserand, A. Soukiassian, L. Xie, X. Q. Pan, T. Dekorsy, D. G. Schlom, and A. Fainstein. Acoustic confinement phenomena in oxide multifunctional nanophononic devices. Phys. Rev. Materials **2**, 106002 (2018).

[21] N. D. Lanzillotti-Kimura, A. Fainstein, C. A. Balseiro, and B. Jusserand, Phonon engineering with acoustic nanocavities: Theoretical considerations on phonon molecules, band structures, and acoustic Bloch oscillations, Phys. Rev. B **75**, 024301 (2007).

[22] A. S. Kuznetsov, D. H. O. Machado,K. Biermann, and P. V. Santos, Electrically Driven Microcavity Exciton-Polariton Optomechanics at 20 GHz, Physical Review X **11**, 021020 (2021).

[23] M. Aspelmeyer, T. J. Kippenberg, and F. Marquardt, Cavity Optomechanics, Rev. Mod. Phys. **86**, 1391 (2014).

[24] Villafañe, V., P. Sesin, P. Soubelet, S. Anguiano, A. E. Bruchhausen, G. Rozas, C. Gomez Carbonell, A. Lemaître, and A. Fainstein, Optoelectronic forces with quantum wells for cavity optomechanics in GaAs/AlAs semiconductor microcavities, Physical Review B **97**, 195306 (2018).

[25] C. Baker, W. Hease, D.-T. Nguyen, A. Andronico, S. Ducci, G. Leo, and I. Favero, Photoelastic coupling in gallium arsenide optomechanical disk resonators, Opt. Express **22**, 14072 (2014).

[26] C. Metzger, I. Favero, A. Ortlieb, and K. Karrai, Optical self cooling of a deformable Fabry-Perot cavity in the classical limit, Phys. Rev. B **78**, 035309 (2008).

[27] A. A. Reynoso, G. Usaj, D. L. Chafatinos, F. Mangussi, A. E. Bruchhausen, A. S. Kuznetsov, K. Biermann, P. V. Santos, and A. Fainstein, Optomechanical parametric oscillation of a quantum light-fluid lattice, Phys. Rev. B **105**, 195310 (2022).

[28] I. Carusotto, and C. Ciuti, Quantum fluids of light, Reviews of Modern Physics **85**, 299 (2013).

[29] J. Kasprzak, M. Richard, S. Kundermann, A. Baas, P. Jeambrun, J. M. J. Keeling, F. M. Marchetti, M. H. Szymańska, R. André, J. L. Staehli, V. Savona, P. B. Littlewood, B. Deveaud and Le Si Dang. Bose–Einstein condensation of exciton polaritons. Nature, **443** (7110), 409-414 (2006).

[30] M. Wouters, Synchronized and desynchronized phases of coupled nonequilibrium exciton-polariton condensates, Phys. Rev. B **77**, 121302(R) (2008).

[31] P. R. Eastham, Mode locking and mode competition in a nonequilibrium solid-state condensate, Phys. Rev. B **78**, 035319 (2008).

[32] L. D. Landau and E.M. Lifshitz, *Mechanics*, Course of Theoretical Physics Vol.1, (Elsevier, 1976).

[33] I. Kovacic, R. Rand, and S. Mohamed Sah, Mathieu's equation and its generalizations: overview of stability charts and their features, Applied Mechanics Reviews **70**, 020802 (2018).

[34] A. Baas, K. G. Lagoudakis, M. Richard, R. Andre, Le Si Dang, and B. Deveaud-Plédran, Synchronized and Phases of Exciton-Polariton Condensates in the Presence of Disroder, Phys. Rev. Lett. **100**, 170401 (2008)

Correspondence *and requests for materials should be addressed to A.F.*
